# Effects of vutrisiran on cardiac structure and function in patients with transthyretin amyloidosis with cardiomyopathy: secondary outcomes of the HELIOS-B trial

**DOI:** 10.1038/s41591-025-03851-z

**Published:** 2025-08-06

**Authors:** Karola S. Jering, Marianna Fontana, Olivier Lairez, Simone Longhi, Olga Azevedo, Caroline Morbach, Shaun Bender, Patrick Y. Jay, John Vest, Bernard E. Bulwer, Narayana Prasad, Scott D. Solomon, Hicham Skali

**Affiliations:** 1 Cardiovascular Division, Brigham and Women’s Hospital, and Harvard Medical School, Boston, MA USA; 2 National Amyloidosis Centre, Division of Medicine, University College London, Royal Free Hospital, London, UK; 3 Department of Cardiology, Toulouse University Hospital, Toulouse, France; 4 Cardiology Unit, Cardiac Thoracic and Vascular Department, IRCCS Azienda Ospedaliero–Universitaria di Bologna, Bologna, Italy; 5 Cardiology Department, Hospital da Senhora da Oliveira, Guimarães, Portugal; 6 Department of Clinical Research and Epidemiology, Comprehensive Heart Failure Center, University Hospital Würzburg, Würzburg, Germany; 7 Department of Medicine I, University Hospital Würzburg, Würzburg, Germany; 8 Alnylam Pharmaceuticals, Cambridge, MA USA

**Keywords:** Medical research, Outcomes research, Heart failure

## Abstract

In the HELIOS-B randomized clinical trial, the RNA interference therapeutic agent vutrisiran reduced the risk of all-cause mortality and recurrent cardiovascular events among patients with transthyretin amyloidosis with cardiomyopathy (ATTR-CM). In this secondary analysis of HELIOS-B, we evaluated vutrisiran’s effects on echocardiographic measures of cardiac structure and function in patients with ATTR-CM receiving vutrisiran or placebo (*n* = 654, 93% men). At 30 months after treatment, as compared to the placebo group, vutrisiran treatment attenuated increases in mean left ventricular (LV) wall thickness (least squares mean difference: −0.4 mm; 95% confidence interval (CI): −0.8, 0.0; *P* = 0.03) and LV mass index (−10.6 g m^−^^2^; 95% CI: −18.0, −3.3; *P* < 0.01). Vutrisiran treatment also attenuated declines in LV ejection fraction (2.0%; 95% CI: 0.3, 3.7; *P* = 0.02), absolute global longitudinal strain (1.2%; 95% CI: 0.7, 1.7; *P* < 0.01) and LV stroke volume (4.1 ml; 95% CI: 1.7, 6.4; *P* < 0.01), and decreased both the average ratio of early diastolic transmitral flow velocity to early diastolic mitral annular tissue velocity (−2.0; 95% CI: −2.9, −1.2; *P* < 0.01) and the early to late diastolic transmitral flow velocities ratio (−0.3; 95% CI: −0.6, −0.0; *P* = 0.04), as compared to placebo. Consistent with its clinical benefits, these echocardiographic findings indicate favorable effects of vutrisiran on cardiac structure and function in patients with ATTR-CM. ClinicalTrials.gov registration: NCT04153149.

## Abstract (web summary)

In the HELIOS-B randomized trial in patients with transthyretin amyloid cardiomyopathy, treatment with the RNA interference agent vutrisiran, which targets transthyretin, led to improved echocardiographic measures of cardiac structure and function, as compared to placebo.

## Main

Transthyretin amyloidosis with cardiomyopathy (ATTR-CM) is an increasingly recognized cause of heart failure (HF) associated with high morbidity and mortality. ATTR-CM is caused by extracellular accumulation of misfolded transthyretin (TTR) in the form of amyloid fibrils in the heart, leading to restrictive physiology, conduction disease and arrhythmias. Infiltration of the myocardium with amyloid fibrils also underlies the characteristic findings of increased ventricular wall thickness, diastolic dysfunction with elevated filling pressures, biatrial enlargement, decreased global longitudinal strain (GLS) with relative apical sparing and, at advanced stages, impairment of left ventricular ejection fraction (LVEF)^1,2^. Echocardiography is used for diagnostic purposes and in conjunction with clinical status and cardiac biomarkers as a tool to monitor disease progression^3–5^.

Vutrisiran, a subcutaneously administered RNA interference therapeutic agent, targets both wild-type and variant TTR messenger RNA in the hepatocyte for degradation, thereby rapidly knocking down circulating levels of TTR protein^6,7^. In HELIOS-B, vutrisiran significantly decreased rates of cardiovascular events and all-cause death among patients with ATTR-CM compared with placebo, and preserved functional capacity and quality of life^6^. In this analysis, we examined the effects of vutrisiran on echocardiographic measures of cardiac structure and function in patients with ATTR-CM who took part in the HELIOS-B trial.

## Results

### Baseline characteristics

Among the 655 patients enrolled in HELIOS-B, 326 were randomized to vutrisiran and 329 to placebo. Baseline characteristics have been previously reported and were well balanced between treatment groups, with the exception of higher N-terminal pro-B-type natriuretic peptide (NT-proBNP) and troponin I levels in the vutrisiran group than the placebo group in the monotherapy population^6^. Median (interquartile range (IQR)) age was 77 years (45–85), 93% were male, 88% had wild-type ATTR-CM, 91% New York Heart Association (NYHA) functional class ≤II and 67% National Amyloidosis Centre (NAC) stage 1. Among the 76 (12%) patients with variant ATTR-CM, the most common variant was V122I. Median (IQR) NT-proBNP level was 1,920 ng l^−1^ (1,100–3,206). Overall, 40% of patients in the vutrisiran group and 39% of patients in the placebo group were using tafamidis at baseline (tafamidis subgroup); the remaining 395 patients constituted the vutrisiran monotherapy population, in whom tafamidis was initiated in 22% of patients in the vutrisiran group and 21% of patients in the placebo group after a median (IQR) follow-up of 17.6 months (12.2–28.1) (Extended Data Table 1).

Table 1 presents baseline echocardiographic characteristics, which were similar between treatment groups. Left ventricular (LV) wall thickness and LV mass index were increased, and the LV cavity was nondilated. Diastolic parameters were indicative of diastolic dysfunction, as suggested by low early diastolic mitral annular tissue velocities (*e*’), left atrial (LA) enlargement, and elevated early to late diastolic transmitral flow velocities (*E*/*A*) and *E*/*e*’ ratios. Despite preserved LVEF, absolute GLS and LV stroke volume were reduced. Right ventricular (RV) free wall thickness was increased and RV function, as assessed by tricuspid annular systolic myocardial velocity (RV *S*’), was mildly impaired. Baseline echocardiographic parameters according to treatment assignment in the vutrisiran monotherapy population and in the baseline tafamidis subgroup are shown in Table 1.

**Table 1:** Baseline echocardiographic parameters according to treatment assignment

Echocardiographic parameter	Overall population			Monotherapy population		
	*n*	Placebo (*n* = 328)	Vutrisiran (*n* = 326)	*n*	Placebo (*n* = 199)	Vutrisiran (*n* = 196)
LV structure						
Mean LV wall thickness (mm)	645	18.2 (2.7)	18.2 (2.6)	388	18.3 (2.9)	18.2 (2.7)
LVEDD (mm)	642	42.7 (5.9)	42.6 (5.8)	387	42.7 (5.5)	42.9 (5.6)
LVEDV (ml)	630	95.6 (29.0)	93.9 (25.9)	377	92.8 (25.0)	91.1 (25.2)
LVESD (mm)	576	33.7 (6.1)	33.7 (5.7)	343	33.5 (5.8)	33.3 (6.1)
LVESV (ml)	627	43.4 (23.2)	43.0 (21.0)	374	42.3 (20.4)	42.4 (20.2)
LV mass index (g m^−^^2^)	637	180.8 (46.1)	182.1 (44.2)	382	185.7 (49.5)	186.7 (46.8)
LV systolic function						
LVEF (%)	627	55.9 (12.4)	55.6 (12.7)	374	55.7 (12.1)	54.8 (12.6)
Absolute GLS (%)	652	14.0 (3.5)	14.0 (3.5)	393	14.3 (3.5)	14.0 (3.4)
Stroke volume (ml)	616	53.8 (19.0)	50.7 (16.3)	373	55.8 (19.6)	51.2 (16.8)
TDI lateral *s*’ (mm s^−1^)	621	49.4 (16.1)	49.5 (15.8)	375	49.8 (15.7)	50.1 (16.7)
TDI septal *s*’ (mm s^−1^)	630	42.0 (11.8)	41.5 (12.6)	383	42.3 (12.1)	41.6 (13.1)
LV diastolic function						
* E*/*A* ratio	353	1.9 (1.0)	2.1 (1.1)	202	1.9 (1.0)	2.1 (1.1)
* E* wave (mm s^−1^)	644	806.7 (199.3)	804.0 (203.4)	387	802.5 (184.1)	814.1 (212.3)
* A* wave (mm s^−1^)	354	489.0 (229.2)	461.4 (226.3)	202	512.5 (235.6)	488.8 (248.5)
Deceleration time (ms)	627	211.8 (55.5)	214.3 (59.0)	375	203.4 (50.7)	209.7 (57.3)
TDI lateral *e*’ (mm s^−1^)	627	58.5 (20.5)	61.4 (22.6)	380	58.3 (19.8)	61.2 (22.6)
TDI septal *e*’ (mm s^−1^)	632	41.3 (12.1)	42.9 (14.3)	383	42.1 (12.7)	42.6 (14.0)
* E*/*e*’ lateral	620	15.3 (6.3)	14.8 (6.7)	321	15.3 (5.7)	15.1 (7.1)
* E*/*e*’ septal	625	21.1 (8.3)	20.5 (7.9)	377	20.5 (6.9)	20.9 (8.0)
* E*/*e*’ average	612	18.2 (6.1)	17.7 (6.6)	367	18.0 (5.6)	18.0 (6.8)
LA size and function						
LA diameter (mm)	632	41.3 (4.8)	41.7 (5.8)	378	40.9 (4.5)	42.0 (6.1)
LA volume index (ml m^−^^2^)	638	38.4 (10.4)	39.0 (10.3)	383	38.7 (9.7)	40.4 (10.4)
TDI lateral *a*’ (mm s^−1^)	403	42.6 (20.5)	43.2 (22.3)	242	42.7 (19.8)	45.5 (24.1)
TDI septal *a*’ (mm s^−1^)	345	40.8 (19.1)	39.8 (18.2)	203	42.0 (19.1)	42.1 (19.7)
RV and pulmonary pressure						
RV free wall thickness (mm)	457	8.3 (1.9)	8.1 (1.8)	283	8.6 (2.0)	8.4 (1.9)
RV end-diastolic area (cm^2^)	558	22.3 (6.1)	21.6 (5.1)	325	21.4 (5.1)	21.6 (5.1)
RV end-systolic area (cm^2^)	554	13.8 (4.5)	13.0 (3.6)	322	13.4 (3.6)	13.1 (3.7)
RV *S*’ (mm s^−1^)	625	93.5 (27.2)	94.0 (26.6)	374	94.8 (28.1)	93.4 (28.1)
TR velocity (m s^−1^)	535	2.5 (0.4)	2.6 (0.5)	308	2.5 (0.4)	2.5 (0.5)
Maximal IVC diameter (mm)	508	20.2 (5.5)	20.4 (5.3)	311	20.0 (5.2)	20.5 (5.4)

Data are descriptive and presented as mean (s.d.).

*E* wave, peak early diastolic transmitral flow velocity; IVC, inferior vena cava; LVEDD, left ventricular end-diastolic dimension; LVEDV, left ventricular end-diastolic volume; LVESD, left ventricular end-systolic dimension; LVESV, left ventricular end-systolic volume; *s*’, peak systolic mitral annular tissue velocity; TDI, tissue Doppler imaging; TR, tricuspid regurgitation.

### Effect of vutrisiran on echocardiographic parameters

**Fig. 1:**
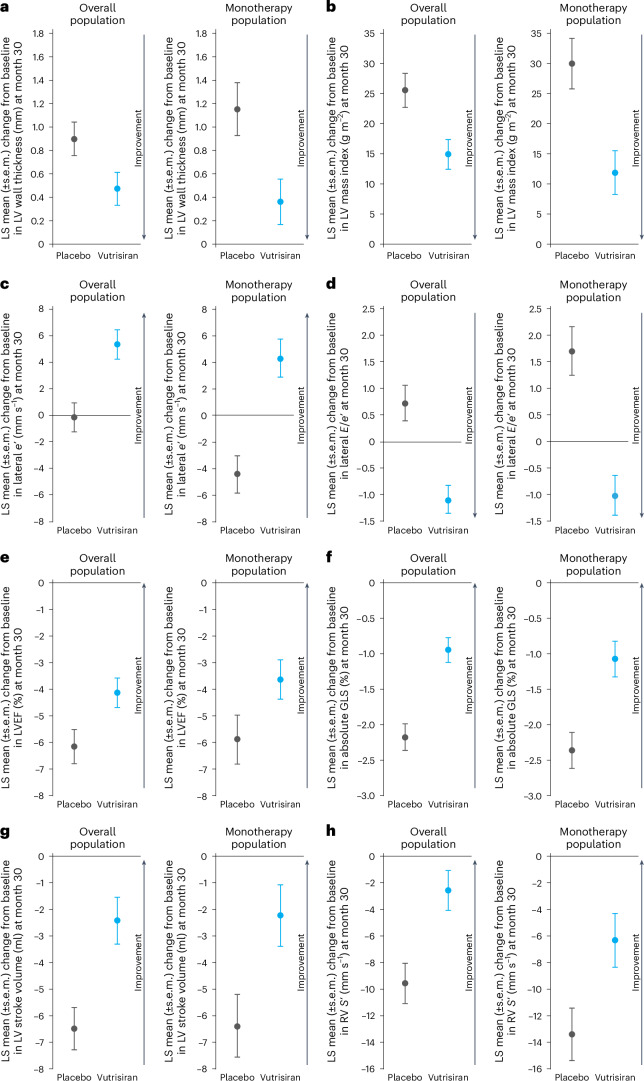
Changes in echocardiographic parameters from baseline to month 30 in the vutrisiran versus placebo groups in the overall and monotherapy populations. **a–h**, LS mean changes in mean LV wall thickness (**a**), LV mass index (**b**), lateral *e*’ velocity (**c**), lateral *E*/*e*’ (**d**), LVEF (**e**), absolute GLS (**f**), LV stroke volume (**g**) and RV *S*’ (**h**) from baseline to month 30 for vutrisiran versus placebo for the overall and monotherapy populations. Dots represent the LS mean change from baseline to month 30 and the error bars represent the s.e.m. Sample size for each echocardiographic parameter can be found in Table 2.

In the placebo group, mean LV wall thickness and LV mass index increased from baseline to month 30. Treatment with vutrisiran attenuated the increases (least squares (LS) mean difference mean LV wall thickness: −0.4 mm; 95% confidence interval (CI): −0.8, 0.0; *P* = 0.03, LV mass index: −10.6 g m^−2^; 95% CI: −18.0, −3.3; *P* = 0.01) (Fig. 1a,b and Table 2). Vutrisiran significantly improved diastolic function across numerous parameters including *e*’ velocity, *E*/*A* and *E*/*e*’ ratios (lateral *e*’ velocity: 5.5 mm s^−1^; 95% CI: 2.4, 8.5; *P* < 0.01; *E*/*A* ratio: −0.3; 95% CI: −0.6, 0.0; *P* = 0.04; average *E*/*e*’ ratio: −2.0; 95% CI: −2.9, −1.2; *P* < 0.01) (Fig. 1c,d). The LA enlarged during follow-up in both treatment groups and vutrisiran did not significantly alter LA size compared with placebo (LA volume index: −0.7 ml m^−^^2^; 95% CI: −2.3, 0.9; *P* = 0.37). LV systolic function, as measured by LVEF, absolute GLS and LV stroke volume, worsened in both treatment groups during follow-up, and vutrisiran significantly and consistently attenuated the decline in these measures of LV systolic function compared with placebo (LVEF: 2.0%; 95% CI: 0.3, 3.7; *P* = 0.02; absolute GLS: 1.2%; 95% CI: 0.7, 1.7; *P* < 0.01; LV stroke volume: 4.1 ml; 95% CI: 1.7, 6.4; *P* = 0.01) (Fig. 1e–g). Vutrisiran also attenuated the decline in RV *S*’ at 30 months (7.0 mm s^−^^1^; 95% CI: 2.8, 11.2; *P* < 0.01) (Fig. 1h). These treatment effects were consistent in sensitivity analyses in which missing data were imputed (Extended Data Table 2).

**Table 2:** Changes in echocardiographic parameters from baseline and treatment differences at month 30

Echocardiographic parameter	Overall population					Monotherapy population				
	*n*	Placebo (*n* = 328)	Vutrisiran (*n* = 326)	Placebo-corrected treatment difference at month 30 (95% CI)	*P* value	*n*	Placebo (*n* = 199)	Vutrisiran (*n* = 196)	Placebo-corrected treatment difference at month 30 (95% CI)	*P* value
LV structure										
Mean LV wall thickness (mm)	455	0.9 (0.1)	0.5 (0.1)	−0.4 (−0.8, 0.0)	0.03	247	1.1 (0.2)	0.4 (0.2)	−0.8 (−1.4, −0.2)	0.01
LVEDD (mm)	442	0.7 (0.3)	0.6 (0.2)	−0.1 (−0.8, 0.6)	0.84	240	0.0 (0.3)	0.3 (0.3)	0.3 (−0.6, 1.2)	0.51
LVEDV (ml)	445	0.4 (1.2)	−1.7 (1.0)	−2.1 (−5.1, 1.0)	0.18	239	0.8 (1.6)	−1.4 (1.4)	−2.2 (−6.2, 1.9)	0.29
LVESD (mm)	375	1.0 (0.3)	−0.2 (0.3)	−1.2 (−2.1, −0.4)	<0.01	195	0.8 (0.4)	−0.2 (0.3)	−1.0 (−2.0, 0.1)	0.07
LVESV (ml)	434	5.9 (0.9)	3.0 (0.8)	−2.9 (−5.3, −0.6)	0.01	232	5.3 (1.2)	2.4 (1.0)	−2.9 (−6.0, 0.1)	0.06
LV mass index (g m^−^^2^)	434	25.4 (2.8)	14.8 (2.5)	−10.6 (−18.0, −3.3)	<0.01	234	29.8 (4.2)	11.8 (3.7)	−18.0 (-28.9, −7.1)	<0.01
LV systolic function										
LVEF (%)	434	−6.2 (0.7)	−4.1 (0.6)	2.0 (0.3, 3.7)	0.02	232	−5.9 (0.9)	−3.6 (0.8)	2.3 (−0.1, 4.6)	0.06
Absolute GLS (%)	471	−2.2 (0.2)	−1.0 (0.2)	1.2 (0.7, 1.7)	<0.01	257	−2.4 (0.3)	−1.1 (0.3)	1.3 (0.6, 2.0)	<0.01
Stroke volume (ml)	427	−6.5 (0.8)	−2.4 (0.9)	4.1 (1.7, 6.4)	<0.01	237	−6.4 (1.2)	−2.2 (1.2)	4.2 (0.9, 7.4)	0.01
TDI lateral *s*’ (mm s^−1^)	426	−1.1 (0.9)	3.5 (0.9)	4.6 (2.1, 7.2)	<0.01	233	−4.0 (1.1)	2.1 (1.2)	6.1 (2.9, 9.3)	<0.01
TDI septal *s*’ (mm s^−1^)	432	−3.0 (0.7)	0.8 (0.7)	3.8 (1.9, 5.6)	<0.01	240	−6.0 (0.8)	−0.6 (0.9)	5.4 (3.0, 7.9)	<0.01
LV diastolic function										
* E*/*A* ratio	175	0.4 (0.1)	0.1 (0.1)	−0.3 (−0.6, 0.0)	0.04	91	0.6 (0.2)	0.1 (0.1)	−0.4 (−0.8, 0.0)	0.05
* E* wave (mm s^−1^)	450	24.3 (10.6)	18.4 (9.3)	−5.8 (−33.5, 21.9)	0.68	245	11.7 (13.7)	5.5 (12.1)	−6.1 (−42.1, 29.8)	0.74
* A* wave (mm s^−1^)	178	−62.2 (18.4)	−6.1 (15.3)	56.1 (8.9, 103.2)	0.02	91	−86.9 (30.0)	−9.5 (20.1)	77.3 (5.4, 149.2)	0.04
Deceleration time (ms)	419	−22.7 (2.9)	−19.9 (2.9)	2.77 (−5.4, 10.9)	0.50	227	−18.0 (3.9)	−6.9 (4.3)	11.1 (−0.3, 22.5)	0.06
TDI lateral *e*’ (mm s^−1^)	438	−0.2 (1.1)	5.3 (1.1)	5.5 (2.4, 8.5)	<0.01	244	−4.5 (1.4)	4.3 (1.4)	8.7 (4.8, 12.6)	<0.01
TDI septal *e*’ (mm s^−1^)	433	−1.6 (0.7)	2.2 (0.7)	3.8 (1.8, 5.8)	<0.01	241	−5.0 (1.0)	0.1 (0.9)	5.2 (2.6, 7.7)	<0.01
* E*/*e*’ lateral	419	0.7 (0.3)	−1.08 (0.3)	−1.8 (−2.7, −1.0)	<0.0001	231	1.7 (0.5)	−1.0 (0.4)	−2.7 (−3.9, −1.6)	<0.0001
* E*/*e*’ septal	415	1.7 (0.4)	−0.6 (0.4)	−2.3 (−3.4, −1.2)	<0.0001	229	3.2 (0.6)	−0.0 (0.5)	−3.2 (−4.7, −1.7)	<0.0001
* E*/*e*’ average	402	1.2 (0.3)	−0.8 (0.3)	−2.0 (−2.9, −1.2)	<0.01	219	2.3 (0.5)	−0.6 (0.4)	−2.9 (−4.1, −1.7)	<0.01
LA size and function										
LA diameter (mm)	422	0.7 (0.3)	0.6 (0.3)	−0.1 (−1.0, 0.7)	0.72	224	0.5 (0.4)	0.8 (0.4)	0.2 (−1.0, 1.4)	0.73
LA volume index (ml m^−^^2^)	457	5.7 (0.6)	5.0 (0.5)	−0.7 (−2.3, 0.9)	0.37	248	5.2 (0.8)	4.7 (0.8)	−0.5 (−2.6, 1.7)	0.67
TDI lateral *a*’ (mm s^−1^)	226	−4.9 (1.5)	−0.9 (1.4)	4.0 (−0.1, 8.0)	0.05	128	−9.7 (2.1)	−4.1 (1.8)	5.6 (0.2, 11.1)	0.04
TDI septal *a*’ (mm s^−1^)	189	−6.4 (1.5)	−3.4 (1.3)	3.0 (−0.8, 6.9)	0.12	107	−11.2 (2.2)	−3.7 (1.7)	7.5 (2.1, 13.0)	0.01
RV and pulmonary pressure										
RV free wall thickness (mm)	255	1.7 (0.0)	1.4 (0.0)	−0.4 (−0.8, 0.0)	0.07	142	1.5 (0.3)	1.0 (0.3)	−0.5 (−1.1, 0.1)	0.10
RV end-diastolic area (cm^2^)	337	0.1 (0.3)	−0.4 (0.4)	−0.5 (−1.5, 0.4)	0.28	178	0.6 (0.4)	−0.1 (0.5)	−0.7 (−2.0, 0.6)	0.30
RV end-systolic area (cm^2^)	319	1.8 (0.3)	1.6 (0.3)	−0.2 (−0.9, 0.6)	0.65	169	1.9 (0.4)	1.4 (0.4)	−0.6 (−1.6, 0.4)	0.25
RV *S*’ (mm s^−1^)	408	−9.6 (1.5)	−2.6 (1.5)	7.0 (2.8, 11.2)	<0.01	226	−13.4 (2.0)	−6.3 (2.0)	7.1 (1.5, 12.7)	0.01
TR velocity (m s^−1^)	334	−0.1 (0.0)	−0.2 (0.0)	0.0 (−0.1, 0.1)	0.60	168	−0.1 (0.0)	−0.1 (0.1)	0.0 (−0.1, 0.1)	0.89
Maximal IVC diameter (mm)	325	1.5 (0.3)	0.2 (0.3)	−1.3 (−2.2, −0.3)	0.01	171	2.1 (0.4)	0.6 (0.4)	−1.6 (−2.8, −0.3)	0.01

Results are reported as the LS mean difference (standard error of the mean (s.e.m.)) derived from repeated measures models with baseline as a covariate and fixed-effect terms including the treatment group, visit, treatment-by-visit interaction, type of ATTR amyloidosis and age group. The overall population also included baseline tafamidis use and treatment-by-baseline tafamidis use interaction as fixed-effect terms.

In the vutrisiran monotherapy population, the treatment effects of vutrisiran, compared with placebo, on LV structure and diastolic and systolic function were similar or greater in magnitude as was seen in the overall population (Fig. 1 and Table 2). In the baseline tafamidis subgroup, the treatment effect of vutrisiran versus placebo was broadly consistent although analyses were underpowered to evaluate treatment differences in this subgroup (Table 3).

**Table 3:** Baseline echocardiographic parameters and changes from baseline to month 30 in the baseline tafamidis subgroup

Echocardiographic parameter	Baseline			LS mean change at month 30		Placebo-corrected treatment difference (95% CI)
	*N*	Placebo (*n* = 129)	Vutrisiran (*n* = 130)	Placebo (*n* = 129)	Vutrisiran (*n* = 130)	
LV structure						
Mean LV wall thickness (mm)	257	18.0 (2.4)	18.2 (2.6)	0.5 (0.2)	0.6 (0.2)	0.1 (−0.1, 0.6)
LVEDD (mm)	255	42.8 (6.5)	42.3 (6.1)	1.7 (0.4)	1.1 (0.4)	−0.6 (−1.7, 0.5)
LVEDV (ml)	253	99.9 (33.9)	98.0 (26.4)	0.08 (1.7)	−2.0 (1.5)	−2.1 (−6.6, 2.4)
LVESD (mm)	233	34.0 (6.5)	34.3 (5.1)	1.3 (0.5)	−0.3 (0.4)	−1.5 (−2.8, −0.3)
LVESV (ml)	253	45.1 (26.9)	43.8 (22.2)	6.9 (1.4)	3.9 (1.1)	−3.0 (−6.5, 0.5)
LV mass index (g m^−2^)	255	173.2 (39.2)	175.3 (39.3)	19.9 (3.6)	18.6 (3.2)	−1.3 (−10.8, 8.3)
LV systolic function						
LVEF (%)	253	56.3 (12.8)	56.9 (12.8)	−6.4 (0.9)	−4.8 (0.8)	1.5 (−0.9, 4.0)
Absolute GLS (%)	259	13.5 (3.4)	13.9 (3.5)	−1.9 (0.3)	−0.8 (0.2)	1.1 (0.4, 1.8)
Stroke volume (ml)	243	50.7 (17.8)	50.0 (15.5)	−6.3 (1.1)	−2.6 (1.3)	3.7 (0.4, 7.1)
TDI lateral *s*’ (mm s^−1^)	246	48.9 (16.8)	48.7 (14.5)	2.9 (1.4)	5.0 (1.5)	2.2 (−1.9, 6.3)
TDI septal *s*’ (mm s^−1^)	247	41.6 (11.1)	41.4 (11.7)	1.1 (1.0)	2.6 (1.0)	1.5 (−1.3, 4.3)
LV diastolic function						
* E*/*A* ratio	151	2.1 (1.1)	2.2 (1.1)	0.2 (0.1)	0.1 (0.1)	−0.1 (−0.5, 0.3)
* E* wave (mm s^−1^)	257	813.0 (221.1)	789.0 (189.3)	38.4 (16.8)	39.0 (14.4)	0.5 (−43.2, 44.2)
* A* wave (mm s^−1^)	152	457.0 (217.8)	425.6 (189.3)	−18.9 (21.6)	−3.0 (23.6)	15.9 (−47.5, 79.3)
Deceleration time (ms)	252	224.3 (60.0)	221.2 (61.0)	−28.3 (4.5)	−36.7 (3.8)	−8.4 (−20.0, 3.2)
TDI lateral *e*’ (mm s^−1^)	247	58.9 (21.5)	61.7 (22.7)	5.9 (1.7)	6.6 (1.7)	0.7 (−4.1, 5.5)
TDI septal *e*’ (mm s^−1^)	249	39.9 (11.2)	43.4 (14.8)	3.1 (1.1)	5.0 (1.2)	1.9 (−1.3, 5.0)
* E*/*e*’ lateral	246	15.4 (7.2)	14.4 (6.1)	−0.6 (0.5)	−1.1 (0.4)	−0.5 (−1.7, 0.7)
* E*/*e*’ septal	248	21.9 (10.1)	20.0 (7.8)	−0.2 (0.6)	−1.3 (0.5)	−1.1 (−2.6, 0.5)
* E*/*e*’ average	245	18.4 (6.8)	17.2 (6.3)	−0.4 (0.5)	−1.2 (0.4)	−0.8 (−1.9, 0.4)
LA size and function						
LA diameter (mm)	254	41.9 (5.3)	41.2 (5.4)	0.9 (0.4)	0.3 (0.4)	−0.6 (−1.7, 0.6)
LA volume index (ml m^−2^)	255	38.0 (11.5)	36.9 (9.8)	6.2 (0.9)	5.2 (0.7)	−1.0 (−3.3, 1.3)
TDI lateral *a*’ (mm s^−1^)	161	42.5 (21.7)	39.7 (19.0)	1.8 (2.0)	3.0 (2.2)	1.1 (−4.7, 7.0)
TDI septal *a*’ (mm s^−1^)	142	39.1 (19.2)	36.6 (15.4)	0.1 (2.0)	−2.6 (1.9)	−2.6 (−8.2, 3.0)
RV and pulmonary pressure						
RV free wall thickness (mm)	174	7.7 (0.2)	7.5 (0.2)	1.9 (0.2)	1.7 (0.2)	−0.3 (−0.9, 0.4)
RV end-diastolic area (cm^2^)	233	23.5 (7.1)	21.6 (5.1)	−0.4 (0.5)	−0.9 (0.5)	−0.5 (−1.9, 1.0)
RV end-systolic area (cm^2^)	232	14.3 (5.4)	12.7 (3.6)	1.6 (0.4)	1.8 (0.4)	0.2 (−0.9, 1.3)
RV *S*’ (mm s^−1^)	251	91.4 (25.6)	95.0 (24.4)	−4.0 (2.4)	2.8 (2.3)	6.8 (0.3, 13.3)
TR velocity (m s^−1^)	227	2.6 (0.4)	2.6 (0.5)	−0.2 (0.0)	−0.2 (0.0)	−0.0 (−0.1, 0.1)
Maximal IVC diameter (mm)	197	20.4 (5.9)	20.2 (5.1)	0.6 (0.5)	−0.2 (0.5)	−0.8 (−2.3, 0.7)

Results are reported as the LS mean difference (s.e.m.) derived from repeated measures models with baseline as a covariate and fixed-effect terms including the treatment group, visit, treatment-by-visit interaction, type of ATTR amyloidosis and age group. The overall population also included baseline tafamidis use and treatment-by-baseline tafamidis use interaction as fixed-effect terms.

Both in the overall population and in the vutrisiran monotherapy population, the effects of vutrisiran on key echocardiographic parameters including mean LV wall thickness, LV mass index, *E*/*A* and *E*/*e*’ ratios, GLS, LVEF and LV stroke volume were consistent across prespecified subgroups (Supplementary Figs. 1–7).

### Temporal changes according to treatment assignment

**Fig. 2:**
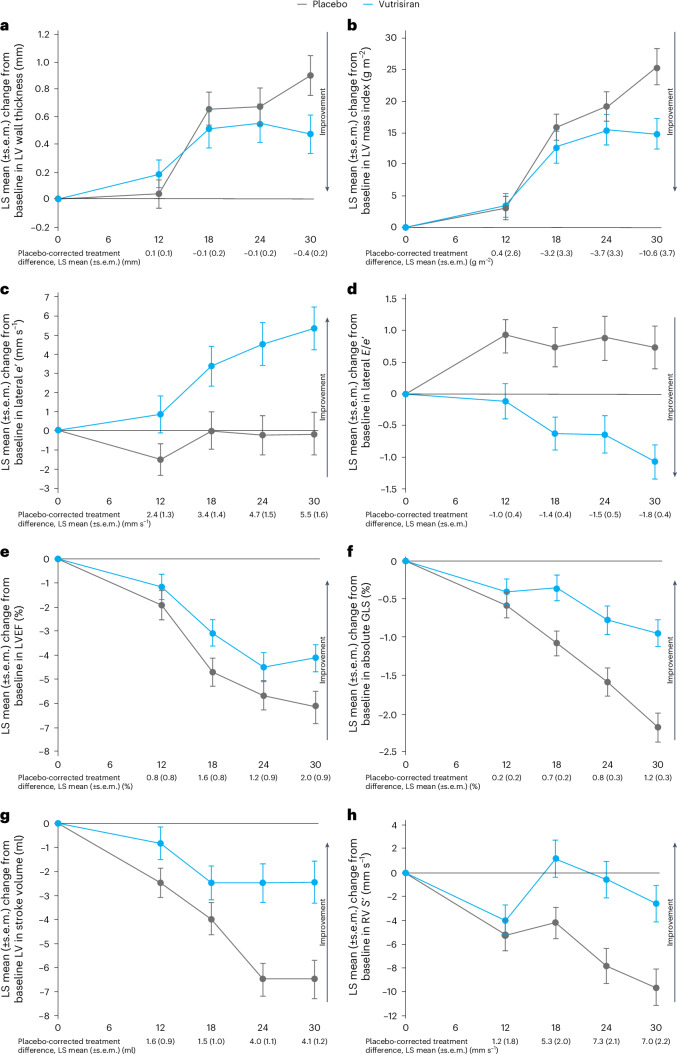
Effect of vutrisiran compared with placebo on echocardiographic parameters over time in the overall population. **a–h**, LS mean changes over time in mean LV wall thickness (**a**), LV mass index (**b**), lateral *e*’ velocity (**c**), lateral *E*/*e*’ (**d**), LVEF (**e**), absolute GLS (**f**), LV stroke volume (**g**) and RV *S*’ (**h**) according to treatment assignment in the overall population. Error bars represent the s.e.m. Sample size for each echocardiographic parameter can be found in Table 2.

**Extended Data Fig. 1:**
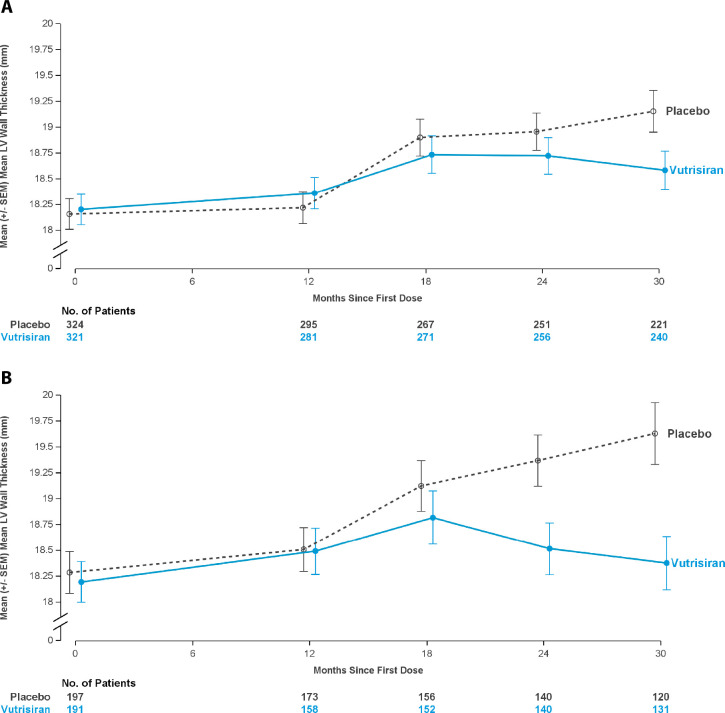
Effect of Vutrisiran on Mean Left Ventricular Wall Thickness Over Time. Mean values (±SEM) during follow-up are shown according to treatment assignment in the (**a**) overall (n = 645) and (**b**) vutrisiran monotherapy population (n = 388). Error bars represent the standard error of the mean.

**Extended Data Fig. 2:**
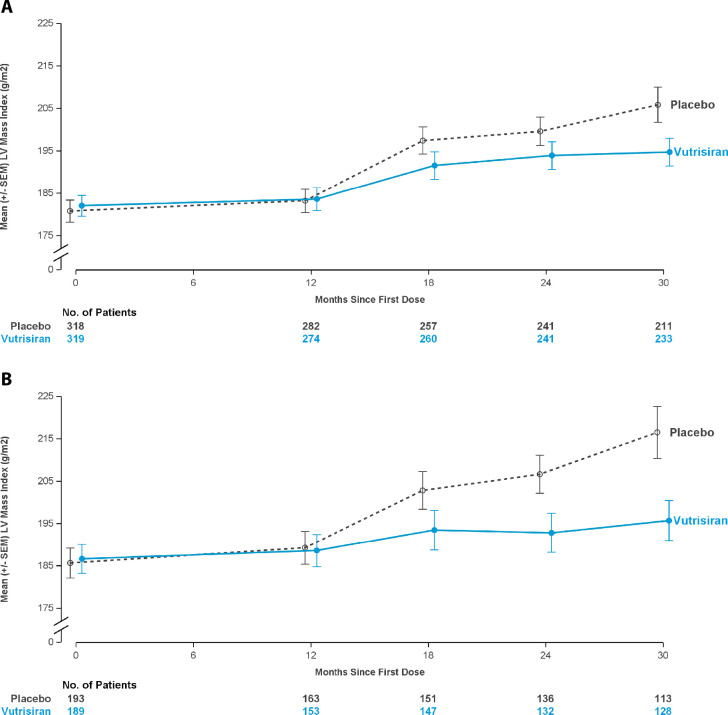
Effect of Vutrisiran on Left Ventricular Mass Index Over Time. Mean values (±SEM) during follow-up are shown according to treatment assignment in the (**a**) overall (n = 637) and (**b**) vutrisiran monotherapy population (n = 382). Error bars represent the standard error of the mean.

**Extended Data Fig. 3:**
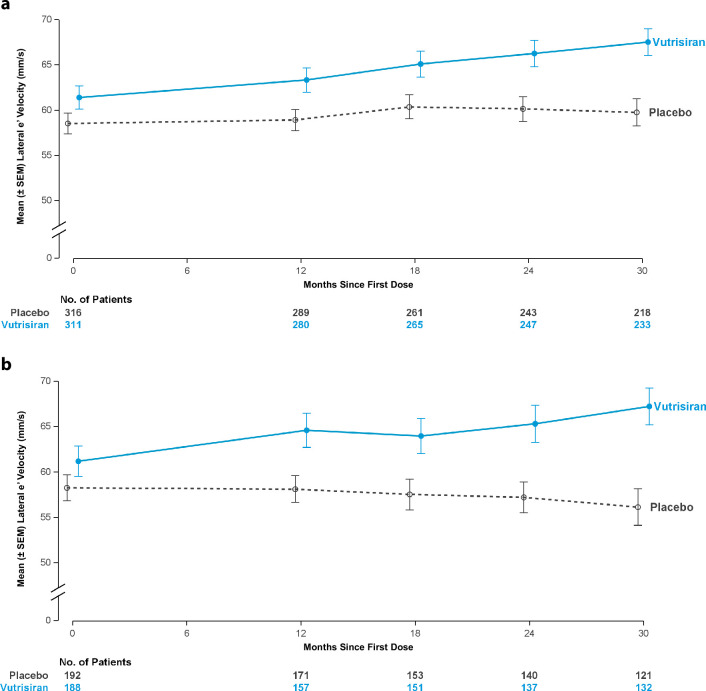
Effect of Vutrisiran on Lateral Early Diastolic Mitral Annular Tissue Velocity (e’) Over Time. Mean values (±SEM) during follow-up are shown according to treatment assignment in the (**a**) overall (n = 627) and (**b**) vutrisiran monotherapy population (n = 380). Error bars represent the standard error of the mean.

**Extended Data Fig. 4:**
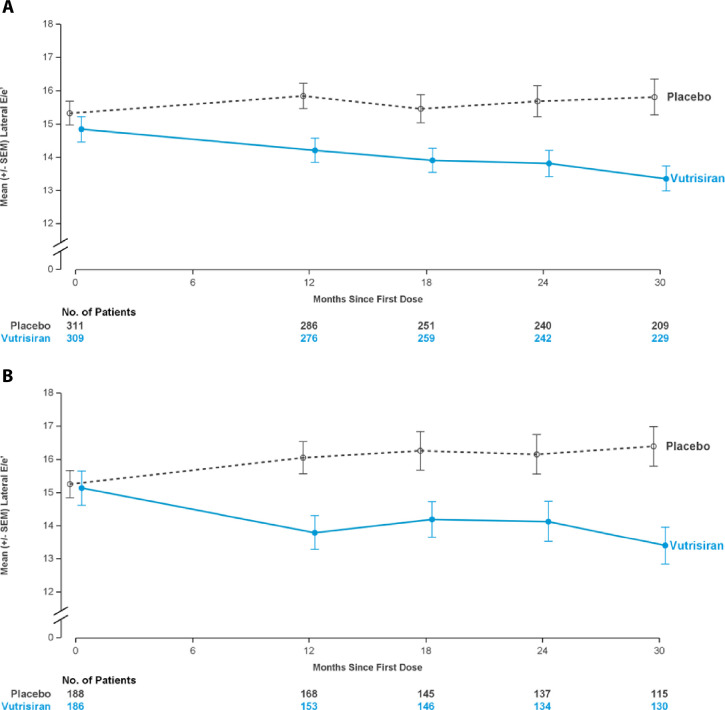
Effect of Vutrisiran on the Ratio of Early Diastolic Transmitral Flow Velocity to Lateral Early Diastolic Mitral Annular Tissue Velocity (E/e’) Over Time. Mean values (±SEM) during follow-up are shown according to treatment assignment in the (**a**) overall (n = 620) and (**b**) vutrisiran monotherapy population (n = 321). Error bars represent the standard error of the mean.

**Extended Data Fig. 5:**
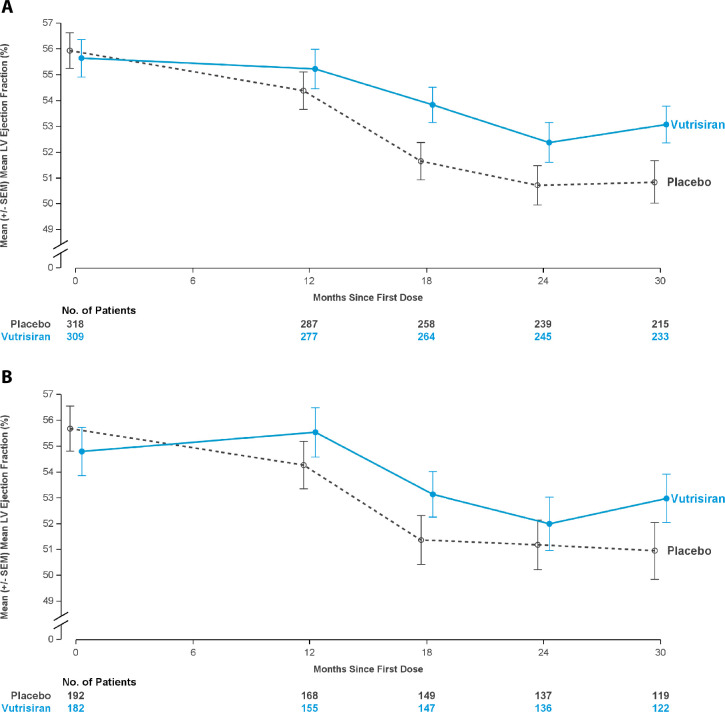
Effect of Vutrisiran on Left Ventricular Ejection Fraction Over Time. Mean values (±SEM) during follow-up are shown according to treatment assignment in the (**a**) overall (n = 627) and (**b**) vutrisiran monotherapy population (n = 374). Error bars represent the standard error of the mean.

**Extended Data Fig. 6:**
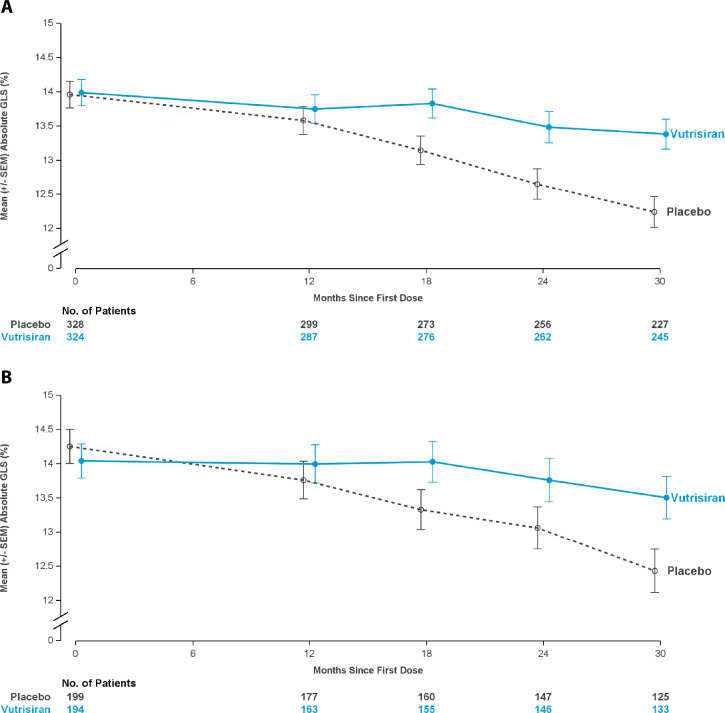
Effect of Vutrisiran on Absolute Global Longitudinal Strain Over Time. Mean values (±SEM) during follow-up are shown according to treatment assignment in the (**a**) overall (n = 652) and (**b**) vutrisiran monotherapy population (n = 393). Error bars represent the standard error of the mean.

**Extended Data Fig. 7:**
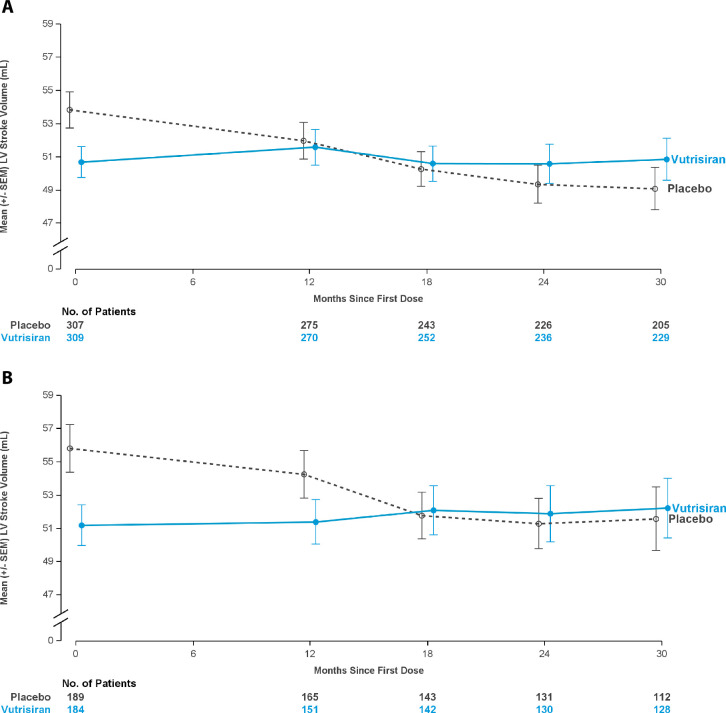
Effect of Vutrisiran on Left Ventricular Stroke Volume Over Time. Mean values (±SEM) during follow-up are shown according to treatment assignment in the (**a**) overall (n = 616) and (**b**) vutrisiran monotherapy population (n = 373). Error bars represent the standard error of the mean.

**Extended Data Fig. 8:**
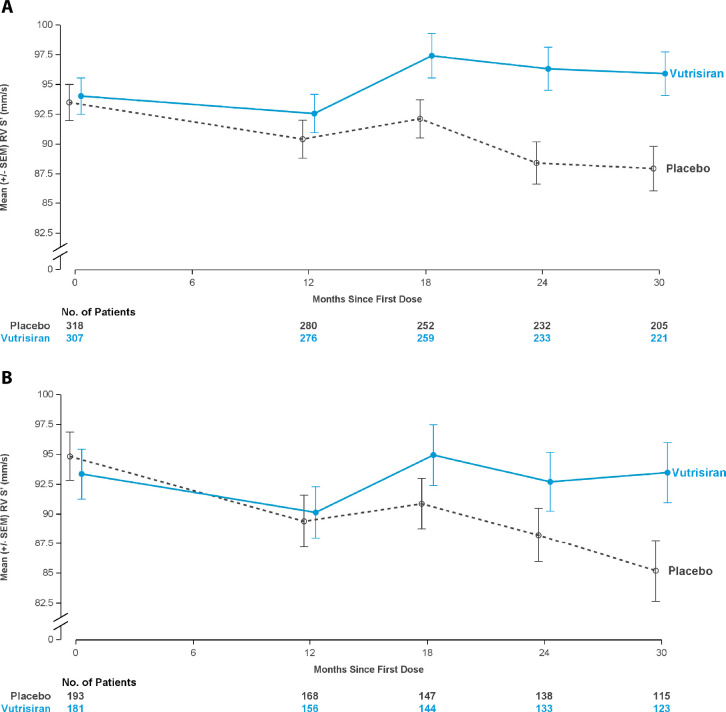
Effect of Vutrisiran on Tricuspid Annular Systolic Myocardial Velocity (RV S’) Over Time. Mean values (±SEM) during follow-up are shown according to treatment assignment in the (**a**) overall (n = 625) and (**b**) vutrisiran monotherapy population (n = 374). Error bars represent the standard error of the mean.

In both treatment groups, mean LV wall thickness and LV mass index increased steadily during follow-up, with attenuation in this increase in the vutrisiran arm that was apparent by month 30 (Fig. 2a,b and Extended Data Figs. 1 and 2). While *E*/*e*’ rose (worsened) in the placebo group during follow-up, it declined (improved) in the vutrisiran group, driven largely by an increase in *e*’ velocity (Fig. 2c,d and Extended Data Figs. 3 and 4). Significant between-group differences in *E*/*e*’ were observed as early as 12 months. Between-group differences in LV systolic function were observed by 18 months with vutrisiran attenuating the progressive worsening in LVEF, GLS and LV stroke volume observed in the placebo group (Fig. 2e–g and Extended Data Figs. 5–7). Similarly, vutrisiran attenuated the decline in RV *S*’ with significant between-group differences observed at 18 months (Fig. 2h and Extended Data Fig. 8).

## Discussion

In this prespecified analysis of the HELIOS-B trial, vutrisiran positively affected echocardiographic measures of cardiac structure and function, improved diastolic function, and significantly attenuated declines in LV systolic function and increases in LV wall thickness and LV mass index over 30 months. The magnitude of the treatment effect of vutrisiran compared with placebo was similar or greater in the vutrisiran monotherapy population. Significant improvement in diastolic function with vutrisiran emerged early, followed by significant favorable effects on LV systolic function and cardiac structure.

Owing to advances in non-invasive diagnostic imaging modalities, increased awareness of ATTR-CM as a cause of HF and growing use of disease-modifying therapies, current patients with ATTR-CM are often identified earlier and have less advanced disease at the time of diagnosis compared with historic cohorts^8^. Concordant with this temporal trend, all-cause mortality was substantially lower in HELIOS-B as compared with previous randomized trials of patients with ATTR-CM including ATTR-ACT and ATTRibute-CM^6,9,10^. While HELIOS-B enrolled patients with a wide range of disease severity, on average, patients also had a better functional status and less advanced LV systolic dysfunction compared with patients enrolled in ATTR-ACT (mean LVEF 56% versus 49%; global longitudinal strain −14% versus −9%; LV stroke volume 52 ml versus 46 ml). These observations mirror findings from the NAC, where patients with ATTR-CM exhibited less pathologic remodeling, less biventricular systolic dysfunction and lower mortality compared with patients referred during earlier periods^9^. The clinical and echocardiographic characteristics of patients enrolled in HELIOS-B were, however, representative of those of contemporary patients with ATTR-CM. Most patients had wild-type ATTR-CM, with preserved LVEF but mildly reduced absolute GLS and stroke volume, mild LA enlargement, diastolic dysfunction and moderately increased LV wall thickness^8^.

ATTR-CM is a progressive disease, characterized on echocardiography by gradual increases in LV wall thickness and progressive deterioration of LV systolic and diastolic function as amyloid infiltrates the myocardium, distorts normal tissue architecture, increases chamber stiffness and causes myocyte injury^3,5^. In HELIOS-B, vutrisiran attenuated these increases in LV wall thickness and LV mass index when compared with placebo over 30 months. As LV mass has been correlated with amyloid burden, attenuating increases in LV mass index likely reflects diminished amyloid deposition in the myocardium with vutrisiran therapy^11^. The impact of TTR knockdown on LV mass has been consistently observed in prior studies of patisiran and vutrisiran in patients with variant ATTR amyloidosis with polyneuropathy, who commonly have cardiac involvement, and of patisiran in patients with ATTR-CM^12–14^. In HELIOS-B, between-group differences in LV wall thickness and LV mass index were observed at 30 months. The rate of increase in LV wall thickness was modest as HELIOS-B enrolled predominantly patients with wild-type ATTR-CM, who are known to exhibit slower and less concentric remodeling as compared with patients with variant ATTR-CM, particularly V122I^5^. It is therefore expected that effects on cardiac structure emerged later.

Diastolic dysfunction is a hallmark of ATTR-CM and plays a crucial role in disease pathophysiology and evolution. In the placebo group, diastolic function deteriorated during follow-up. In contrast, vutrisiran significantly improved several indices of diastolic function including *e*’ velocity, *E*/*e*’ and *E*/*A* ratios. These effects of vutrisiran on diastolic function emerged early with statistically significant between-group differences for *E*/*e*’ observed as early as 12 months, earlier than expected from the time course of effects on LV structure. While vutrisiran had beneficial effects on multiple measures of diastolic function, the effect on LA size compared with placebo was not statistically significant. Extensive amyloid infiltration of the atria, as shown by histology and cardiac magnetic resonance imaging, causes poor compliance and impaired LA contractile function^15–17^. Poor compliance limits atrial remodeling in response to rising ventricular filling pressures. Interestingly, peak late diastolic mitral annular tissue velocity (*a*’) and peak late diastolic transmitral flow velocity (*A* wave) decreased in the placebo group but remained stable in the vutrisiran group during follow-up. Whether this represents preservation of LA contractile function, favorable effects on LV compliance or a combination of both deserves further exploration with more sensitive measures of LA function.

LV systolic function, an important and independent predictor of mortality in patients in ATTR-CM, deteriorated during follow-up. The rate of decline in LVEF and absolute GLS in the placebo group was comparable to that observed in the placebo arm of ATTR-ACT and in retrospective cohort studies^5,9,18^. Compared with placebo, vutrisiran attenuated declines in LV systolic function across multiple indices with significant between-group differences observed as early as 18 months. The magnitude of the treatment effect of vutrisiran on measures of LV systolic function including LVEF, GLS and LV stroke volume was comparable with that of tafamidis in ATTR-ACT although comparisons are difficult to draw between the two trials owing to differences in patient characteristics, disease severity and background treatment^18^. Notably, tafamidis attenuated worsening in the *E*/*e*’ ratio in ATTR-ACT whereas vutrisiran improved the *E*/*e*’ ratio in HELIOS-B compared with placebo^18^. As measures of LV systolic function including GLS have been correlated with amyloid burden, inhibiting further amyloid deposition in the myocardium may underlie the favorable effects of vutrisiran on LV systolic function^19^. The treatment effects of vutrisiran on systolic and diastolic function also complement its favorable effects on NT-proBNP, a marker of ventricular wall stress and important prognostic marker in ATTR-CM, and cardiac troponin, a marker of myocardial injury^6^. The effects of vutrisiran on cardiac structure and function were similar or larger in magnitude in the vutrisiran monotherapy population.

The beneficial effects of vutrisiran on echocardiographic measures of cardiac structure and function are clinically meaningful as they were observed despite substantial background use of tafamidis (40% at baseline with an additional 22% drop in in the monotherapy population during follow-up)^6^ (Extended Data Table 1). The echocardiographic parameters positively affected by vutrisiran including *E*/*e*’, GLS, LVEF and stroke volume are known to be prognostically important in patients with ATTR-CM^20,21^. Differences of the magnitude observed with vutrisiran in HELIOS-B in these parameters have been shown to be independently associated with mortality. Furthermore, declines in LV stroke volume index over 12 months as well as worsening severity of mitral and tricuspid regurgitation in untreated patients with ATTR-CM have been independently associated with a higher risk of all-cause mortality^5^. These findings suggest that declines in the effective forward stroke volume as the final common pathway in ATTR-CM portend a worse prognosis, as would be expected in a patient population with restrictive physiology. The beneficial effects of vutrisiran on cardiac function, including on LV stroke volume, may therefore contribute to improved outcomes. Furthermore, the time course of beneficial effects on LV structure and function provides a mechanistic explanation for the impact soon after vutrisiran treatment initiation on reducing the risk of worsening heart failure^22^. The findings support early use of vutrisiran in patients with ATTR-CM regardless of background therapy.

The present findings should be interpreted in the context of study limitations. Although tafamidis was used in 40% of patients at baseline, the study was not powered to estimate treatment effects in the tafamidis subgroup. Racial diversity and enrollment of women were limited in HELIOS-B, reflecting the reported demographic characteristics of patients with ATTR-CM^2,8^. Consistent with the prevalence of wild-type ATTR-CM and male predominance, a lower proportion of patients with variant ATTR-CM and women were enrolled, which likewise limited the power to estimate treatment effects in these subgroups^8^. Information on severity of valvular disease was not collected.

In conclusion, in this current population of patients with ATTR-CM, vutrisiran had beneficial impact across all domains of cardiac structure, systolic and diastolic function consistent with its beneficial effects on clinical outcomes, biomarkers and health status. The beneficial effects of vutrisiran on cardiac structure and function likely underlie the reduced risk of cardiovascular events and all-cause mortality, as well as outpatient worsening HF. These favorable effects on cardiac structure and both systolic and diastolic function, despite extensive use of background therapy, further support its use as a disease-modifying therapy for patients newly diagnosed with ATTR-CM and those progressing on stabilizing therapies.

## Methods

### HELIOS-B study design and population

The design, study protocol, statistical analysis plan and primary results of HELIOS-B have been previously reported^6^. Briefly, HELIOS-B was a double-blind, randomized, placebo-controlled trial testing the efficacy and safety of vutrisiran (25 mg administered subcutaneously every 12 weeks) versus placebo in patients with ATTR-CM. Patients aged from 18 to 85 years were eligible if they had a diagnosis of either variant or wild-type ATTR-CM established on the basis of tissue biopsy or validated scintigraphy-based diagnostic criteria with evidence of cardiac involvement (interventricular septal wall thickness >12 mm on echocardiography) and a clinical history of symptomatic HF. Additional inclusion criteria were NT-proBNP level >300 pg ml^−1^ (>600 pg ml^−1^ in patients with atrial fibrillation) and <8,500 pg ml^−1^, and 6-min walk distance ≥150 m. At baseline, patients were either receiving tafamidis for ATTR-CM at the dose approved within their country or were not receiving tafamidis, with no active plan to start tafamidis during the first 12 months after randomization. Key exclusion criteria were NYHA functional class IV or NYHA III with NAC ATTR stage 3, prior use of TTR-lowering therapies and estimated glomerular filtration rate <30 ml per min per 1.73 m^2^. A complete list of inclusion and exclusion criteria is provided in the primary publication^6^. The ethics committee at each participating site approved the study protocol (see Supplementary Appendix for full list) and patients provided written informed consent. Medidata Rave EDC v.2024.1.1 was used for case report form data collection.

### Study procedures and echocardiographic methods

Certified sonographers at each site performed serial echocardiograms according to a standardized, prespecified protocol at baseline and months 12, 18, 24 and 30. Echocardiographic images were transferred to the Cardiovascular Imaging Core Laboratory (Brigham and Women’s Hospital, Boston, MA, USA) for analysis. Echocardiographic measurements were performed using commercially available software (Us2.ai v.1.4 and v.2.0) by analysts blinded to clinical characteristics of study participants, treatment assignment and temporal sequence. Quantitative measurements were performed in accordance with American Society of Echocardiography guidelines^23,24^. Laboratory-wide intra- and interobserver variability for key measures of cardiac structure and function have been previously reported^25–27^; amongst analysts involved in this study the coefficient of variation was ≤16% and intraclass correlation ≥0.72.

Mean LV wall thickness was computed as the average of interventricular septal and posterior wall thickness in diastole and relative wall thickness as ×2 posterior wall thickness in diastole/left ventricular end-diastolic diameter. LV mass was calculated from linear dimensions using Devereux’s formula and indexed to body surface area. LV volumes and LVEF were derived using the modified biplane Simpson’s method^23^. LV stroke volume was calculated as π × (LV outflow tract diameter/2)^2^ × LV outflow tract velocity time integral. LV GLS was measured on the Us2.ai platform^28^. LA volume was measured from apical two- and four-chamber views at end systole. Mitral inflow (*E* and *A* wave velocities) was assessed using pulsed-wave Doppler from the apical four-chamber view and tissue velocities (*e*’, *a*’ and *s*’) were measured from the septal and lateral aspects of the mitral annulus. RV free wall thickness was measured in the subcostal views and RV function was assessed using RV *S*’.

Changes from baseline to month 30 in mean LV wall thickness and GLS were prespecified as exploratory endpoints in HELIOS-B. Changes in other echocardiographic parameters were not prespecified and as such are considered exploratory.

### Statistical analysis

Data were summarized as frequency (percentage) for categorical variables and mean ± s.d. or median (IQR) for continuous variables, depending on their distribution. Changes in echocardiographic parameters from baseline to month 30 were evaluated using repeated measures models with an unstructured covariance matrix. The corresponding baseline echocardiographic parameter was included as a covariate and treatment group, visit (baseline, month 12, 18, 24 or 30), treatment-by-visit interaction, type of ATTR amyloidosis (wild-type versus variant) and age group (<75 versus ≥75 years) were included as fixed-effect terms. Changes in echocardiographic parameters were analyzed both in the overall population as well as in the vutrisiran monotherapy population, defined as patients not taking tafamidis at baseline, as previously described^6^. In the overall population, baseline tafamidis use and treatment-by-baseline tafamidis use interaction were also included as fixed-effect terms. Missing data were not imputed (the number of missing values is reported in Supplementary Table 1). A sensitivity analysis using a pattern mixture model was performed to assess the robustness of the repeated measures model results to the possible violation of the missing at random (MAR) missingness assumption. For this, patients were classified into four patterns for missing echocardiographic data during the double-blind period:

1. Patients who died (including those who underwent heart transplantation or LV assist device placement) before the month 30 visit: the missing change from baseline values at a postbaseline visit were imputed as sampling with replacement from the worst 10% of observed change from baseline values from all patients at the same visit and in the same treatment group and same baseline tafamidis group, capped by the worst possible change for the patient (that is 0 – baseline value).
2. Patients in the vutrisiran arm who were not in Pattern 1 and had missing visits within 126 days of the last dose of vutrisiran: the missing change from baseline values was considered MAR and was imputed using multiple imputation (MI) estimated from vutrisiran patients with nonmissing data collected on treatment (that is, data on visits within 126 days from last dose) in the same baseline tafamidis group. The window of 126 days was chosen as ×1.5 the dosing interval of 84 days.
3. Patients in the vutrisiran arm who were not in Pattern 1 and had missing visits that were more than 126 days after the last dose of vutrisiran:
  (i) If there were sufficient (that is, at least 10) retrieved dropouts at month 30 in the same baseline tafamidis group (that is, vutrisiran patients who had discontinued treatment but still had assessments that were more than 126 days after the last dose), the missing data were imputed using data from these retrieved dropouts.
  (ii) If there were insufficient retrieved dropouts, missing change from baseline values were assumed to be missing not at random and were imputed (using data from placebo patients in the same baseline tafamidis group) using the copy reference approach.
4. Patients in the placebo arm who were not in Pattern 1 and had missing data for other reasons:
  (i) If there were sufficient (that is, at least 10) retrieved dropouts at month 30 in the same baseline tafamidis group (that is, placebo patients who had discontinued study treatment but still had assessments that were more than 126 days from the last dose), the missing data were imputed using data from these retrieved dropouts.
  (ii) If there were insufficient retrieved dropouts, missing change from baseline values were considered as MAR and imputed using MI estimated from all placebo patients in the same baseline tafamidis group.

Imputed change from baseline from any of the methods was capped by the worst possible change of the patient (0 – baseline value). For patients in Pattern (1), the imputation details were as described above. For patients in Pattern (2) and (4ii), all missing data for the placebo arm and missing data for the vutrisiran arm during the on-treatment period (that is, assessments within 126 days of the last dose of study drug) were imputed using MI under the MAR assumption. As the pattern of missing data within patients may be nonmonotone, multiple imputation was conducted separately by treatment arm and baseline tafamidis use group using the Markov Chain Monte Carlo method. For each treatment arm or baseline tafamidis use group, the imputation model included type of ATTR amyloidosis (wild-type versus variant), NYHA class (I or II versus III), age at randomization (<75 versus ≥75 years), NT-proBNP (≤3,000 ng l^−1^ versus >3,000 ng l^−1^), baseline value and all postbaseline change from baseline values at protocol specified visits.

For imputation using the retrieved dropouts (Patterns (3i) and (4i)), the multiple imputation approach described in ref. ^29^ was adopted.

Missing values were imputed 100 times to generate 100 complete datasets using the procedures described above (via MI, copy reference or other methods described previously). An analysis of covariance (ANCOVA) model was fitted to each imputed dataset for the change from baseline in select echocardiographic parameters at month 30. The ANCOVA model included the baseline echocardiographic parameter as a covariate and treatment arm, baseline tafamidis use, treatment-by-baseline tafamidis use interaction, ATTR disease type and age group as factors. For the vutrisiran monotherapy subgroup analysis, baseline tafamidis use and treatment-by-baseline tafamidis use interaction were removed from the model.

The LS mean and standard error estimated from the ANCOVA model fit to each imputed dataset were combined by applying Rubin’s rules to produce inferential results including the treatment difference in LS means and 95% CI for the treatment difference^29–31^. Statistical significance was assessed using a two-sided alpha level of 0.05 without adjustment for multiplicity. All analyses were performed using SAS v.9.4.

### Reporting summary

Further information on research design is available in the Nature Portfolio Reporting Summary linked to this article.

## Online content

Any methods, additional references, Nature Portfolio reporting summaries, source data, extended data, supplementary information, acknowledgements, peer review information; details of author contributions and competing interests; and statements of data and code availability are available at 10.1038/s41591-025-03851-z.

## Supplementary information

Supplementary Information Supplementary Figs. 1–8 and Table 1.

Reporting Summary

## Figures and Tables

**Extended Data Table 1:**
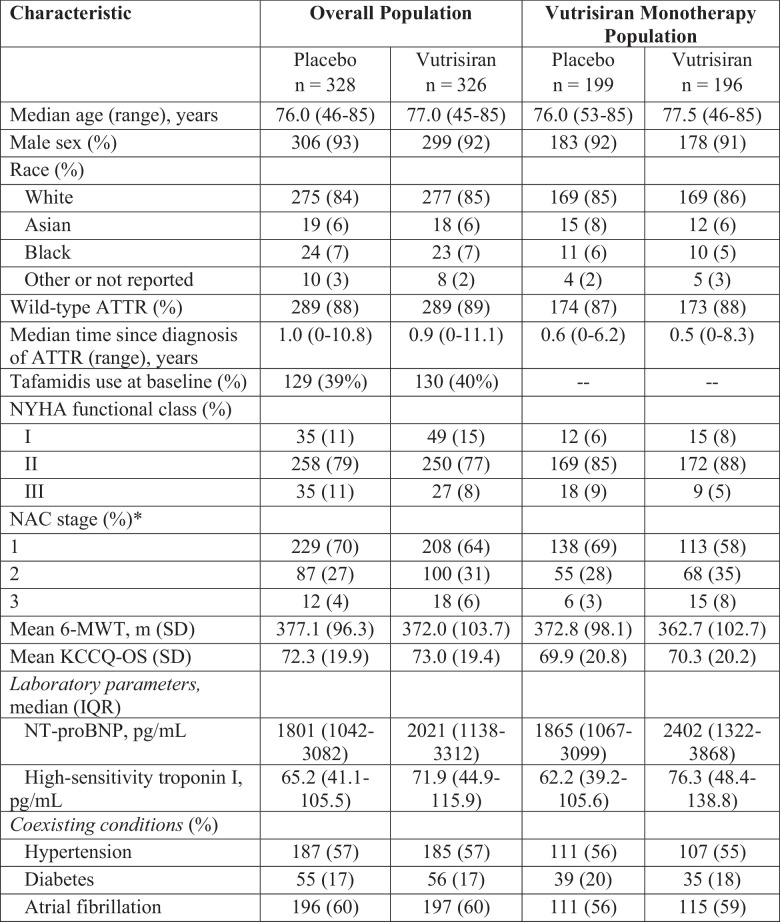
Baseline Demographics and Disease Characteristics According to Treatment Assignment and Underlying Tafamidis Treatment

Values are n (%), mean ± SD, or median (IQR). Race was self-reported. *NAC stage classifies patients according to NT-proBNP and eGFR levels: Stage 1 (lower risk) is defined as NT-proBNP ≤3000 pg/mL and eGFR ≥45 mL/min/1.73 m^2^; stage 2 (intermediate risk) includes patients not meeting stage 1 or 3 criteria; stage 3 (higher risk) is defined as NT-proBNP 3000 pg/mL and eGFR 45 mL/min/1.73 m^2^. 6-MWT – 6-minute walk test, ATTR – transthyretin amyloidosis, eGFR – estimated glomerular filtration rate, IQR – interquartile range, KCCQ-OS – Kansas City Cardiomyopathy Questionnaire-Overall Summary; NAC – National Amyloidosis Centre; NT-proBNP – N-terminal pro-B-type natriuretic peptide, NYHA – New York Heart Association, SD – standard deviation.

**Extended Data Table 2:**
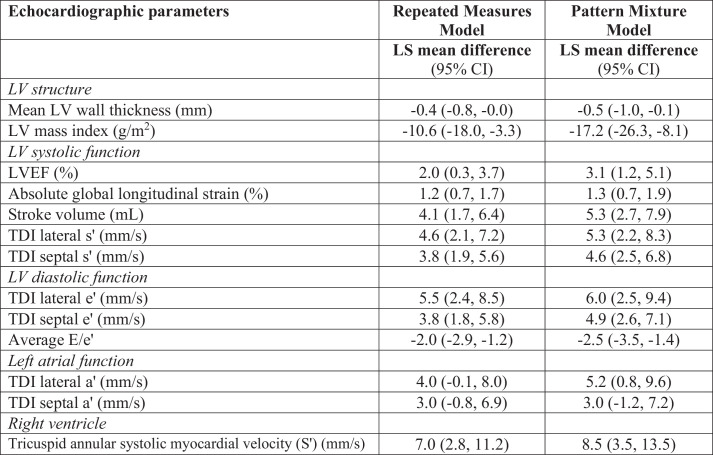
Placebo-Corrected Treatment Difference at Month 30 with and without Imputation for Missing Data for Selected Echocardiographic Variables in the Overall Population

a’ – peak late diastolic mitral annular tissue velocity, e’ – peak early diastolic mitral annular tissue velocity, E wave – peak early diastolic transmitral flow velocity, LV – left ventricular, LVEF – left ventricular ejection fraction, s’ – peak systolic mitral annular tissue velocity, TDI – tissue Doppler imaging.

## Data Availability

Access to anonymized individual participant data that support these results will be made available 12 months after study completion and not less than 12 months after the product and indication have been approved in the United States and/or the European Union. Access to data may be declined where there is likelihood a patient could be identified or other feasibility issue, where there is a potential conflict of interest, planned business activities or an actual or potential competitive risk. Data will be provided contingent upon the approval of a research proposal and the execution of a data sharing agreement. Timeframes for data access may vary and can take up to 6 months or more. Requests for access to data can be submitted via the website www.vivli.org. Questions can also be directed to datasharing@alnylam.com.
