# Supplementary material for: Effects of vutrisiran on cardiac structure and function in patients with transthyretin amyloidosis with cardiomyopathy: secondary outcomes of the HELIOS-B trial
Source: Nat Med. 2025 Aug 6;31(10):3560–8. doi: 10.1038/s41591-025-03851-z (PMC12532587; doi:10.1038/s41591-025-03851-z)
Supplement: Supplementary file 1 — Supplementary Figs. 1–8 and Table 1. [file 41591_2025_3851_MOESM1_ESM.pdf]

nature medicine

Supplementary information https://doi.org/10.1038/s41591-025-03851-z

Effects of vutrisiran on cardiac structure and function in patients with transthyretin amyloidosis with cardiomyopathy: secondary outcomes of the HELIOS-B trial

In the format provided by the authors and unedited

SUPPLEMENT

Full list of committees (IRBs/ECs) that approved the HELIOS-B protocol where patients were enrolled

Universitaire de Cardiologie et de Pneumologie de QuÈbec
Administración Nacional de Medicamentos Alimentos y Tecnología Médica (ANMAT)
Advarra- Institutional Biosafety Committee (IBC)
Advarra Institutional Review Board
Agencia Española de Medicamentos y Productos Sanitarios (AEMPS)
AGES PharmMed
Alfred Research and Ethics Unit
ANSM
Autoridade Nacional do Medicamento e Produtos de Sa˙de, I.P. (INFARMED)
BASG/AGES - Institut Zulassung & LiveCycleManagement - LCM/KPPS
Bnai Zion Medical Center EC
CEIC Hospital Universitari de Bellvitge
Central Adelaide Local Health NetworK (CALHN) Human Research Ethics Committees
Central Adelaide Local Health Network Research Office
Centrale Commissie Mensgebonden Onderzoek
CER du CHU de Quebec-Universite Laval
Cleveland Clinic Institutional Review Board
Columbia University Medical Center IRB
Comissão de Ética para a Investigação Clínica - CEIC
Comité de Bioética del Hospital Universitario de la Fundación Favaloro
Comité de Ética de Protocolos de Investigación
ComitÈ de Ètica independiente Instituto Cardiovascular de Buenos Aires
Comite d'Ethique de la recherche de l'Institut Universitaire de Cardiologie et de Pneumologie
Comite d'Ethique de la Recherche du Center de Sante et de Services Sociaux de Rimouski-Neigette
Comité Institucional de Bioética (CIB) de Vía Libre
CPP Sud Mediterranee V
De Videnskabsetiske Komitéer for Region Syddanmark
Direktoratet for Medisinske Produkter DMP
Drug Committee at University Hospital Centre Zagreb
Duke University Health System Institutional Review Board
Egeszsegugyi Tudomanyos Tanacs Klinikai Farmakologiai Etikai Bizottsag
Ethik-Kommission der Ärztekammer Westfalen-Lippe und der Westfälischen Wilhelms-Universität Münster
Ethikkommission der Medizinischen Fakultät Heidelberg
Ethikkommission der Medizinischen Universität Wien
Ethik-Kommission der Medizinischen Universität Wien und des Allgemeinen Krankenhauses der Stadt Wien

Ethik-Kommission der Sächsischen Landesärztekammer
Ethikkommission der Stadt Wien
Eticka komise Fakultni nemocnice Olomouc
Eticka komise IKEM a FTN Fakultni Thomayerova nemocnice
Etikprövningsmyndigheten
European Medicines Agency
Federal Agency for Medicines and Health Products
FundaciÛn Favaloro. Hospital Universitario. ComitÈ de Docencia e InvestigaciÛn
Gesundheitsamt der Stadt Düsseldorf
Halmed
Haradoi Hospital Institutional Review Board
Health Research Authority
Hokubukyushusaiseikai Joint Institutional Review Board
Houston Methodist Research Institute IRB
Kagawa University Hospital IRB
Keio University IRB
Kochi Medical School Hospital Institutional Review Board
Komisja Bioetyczna przy Uniwersytecie Medycznym we Wroclawiu
Kumamoto University Hospital Institutional Review Board
Kurume University Hospital IRB
Kyushu University Hospital Institutional Review Board
Lægemiddelstyrelsen
Läkemedelsverket
Landesdirektion Sachsen
Lithuanian Bioethics Committee
London - Surrey Borders
Mass General Brigham Incorporated
Mater Misericordiae University Hospital
Mayo Clinic Institutional Review Board
Mayo Clinic IRB
Medicines and Healthcare Products Regulatory Agency
Metro South Health Service District Human Research Ethics Committee
MHRA
Ministerie van Volksgezondheid, Welzijn en Sport (VWS)
Ministerio de Salud Instituto Nacional de Salud
Ministry of Health of the Republic of Croatia
Nagoya University Hospital Institutional Review Board
Nara Medical University Hospital IRB
Nara Prefecture General Medical Center IRB
National Cerebral and Cardiovascular Center Institutional Review Board
National Office for Research Ethics Committees
Nemzeti Nepegeszsegugyi és Gyogyszereszeti Kozpont
Osaka University Hospital Institutional Review Board

Partners Human Research Committee
Raad van Bestuur Universitair Medisch Centrum Groningen
Raad van Bestuur Universitair Medisch Centrum Utrecht
Regierung von Oberfranken
Regierungspräsidium Darmstadt
Regierungspräsidium Karlsruhe
Regional komité for medisinsk og helsefaglig forskningsetikk, Sør-Øst-Norge (REK Sør-Øst)
Research Compliance Office, Stanford University
Royal Adelaide Hospital Research Ethics Committee
Samsung Medical Center Institutional Review Board
Samsung Medical Center IRB
Severance Hospital, Yonsei University Health System IRB
Shinshu University Hospital Institutional Review Board
SJH/AMNCH Research Ethics Committee
St Vincent's Ethics and Medical Research Committee
St Vincent's Hospital Human Research Ethics Committee
St Vincent's Hospital Melbourne Human Research Ethics Committee
St. James's Hospital
Staatliches Gewerbeaufsichtsamt Braunschweig
Stanford University Research Compliance Office
State Medicines Controle Agency SMCA
Statni ustav pro kontrolu leciv
The Ethics Committee for Clinical Trials of Medicinal Products
The State Agency of Medicines
Therapeutic Goods Administration
University Health Network Ethics Research Board
University Health Network Research Ethics Board
Urzad Rejestracji Produktów Leczniczych, Wyrobów Medycznych i Produktów Biobójczych
Western Sydney Local Health District Human Research Ethics Committee

Supplementary Figure Legends

Supplementary Figure 1. Subgroup Analysis of the Treatment Effect of Vutrisiran on Mean Left Ventricular Wall Thickness
The forest plot illustrates the treatment effect of vutrisiran versus placebo on mean LV wall thickness at 30 months across prespecified subgroups in (a) the overall population (n=645) and (b) the vutrisiran monotherapy population (n=388).

Supplementary Figure 2. Subgroup Analysis of the Treatment Effect of Vutrisiran on Left Ventricular Mass Index
The forest plot illustrates the treatment effect of vutrisiran versus placebo on left ventricular mass index at 30 months across prespecified subgroups in (a) the overall population (n=637) and (b) the vutrisiran monotherapy population (n=382).

Supplementary Figure 3. Subgroup Analysis of the Treatment Effect of Vutrisiran on the Lateral Early Diastolic Mitral Annular Tissue Velocity (e')
The forest plot illustrates the treatment effect of vutrisiran versus placebo on the lateral early diastolic mitral annular tissue velocity (e') at 30 months across prespecified subgroups in (a) the overall population (n=627) and (b) the vutrisiran monotherapy population (n=380).

Supplementary Figure 4. Subgroup Analysis of the Treatment Effect of Vutrisiran on the Lateral E/e' Ratio
The forest plot illustrates the treatment effect of vutrisiran versus placebo on the lateral E/e' ratio at 30 months across prespecified subgroups in (a) the overall population (n=620) and (b) the vutrisiran monotherapy population (n=321).

Supplementary Figure 5. Subgroup Analysis of the Treatment Effect of Vutrisiran on LVEF
The forest plot illustrates the treatment effect of vutrisiran versus placebo on left ventricular ejection fraction at 30 months across prespecified subgroups in (a) the overall population (n=627) and (b) the vutrisiran monotherapy population (n=374).

Supplementary Figure 6. Subgroup Analysis of the Treatment Effect of Vutrisiran on Absolute Global Longitudinal Strain
The forest plot illustrates the treatment effect of vutrisiran versus placebo on absolute global longitudinal strain at 30 months across prespecified subgroups in (a) the overall population (n=652) and (b) the vutrisiran monotherapy population (n=393).

Supplementary Figure 7. Subgroup Analysis of the Treatment Effect of Vutrisiran on Left Ventricular Stroke Volume
The forest plot illustrates the treatment effect of vutrisiran versus placebo on left ventricular stroke volume at 30 months across prespecified subgroups in (a) the overall population (n=616) and (b) the vutrisiran monotherapy population (n=373).

Supplementary Figure 8. Subgroup Analysis of the Treatment Effect of Vutrisiran on Tricuspid Annular Systolic Myocardial Velocity

The forest plot illustrates the treatment effect of vutrisiran versus placebo on tricuspid annular systolic myocardial velocity at 30 months across prespecified subgroups in (a) the overall population (n=625) and (b) the vutrisiran monotherapy population (n=374).

Supplementary Figures

Supplementary Figure 1.

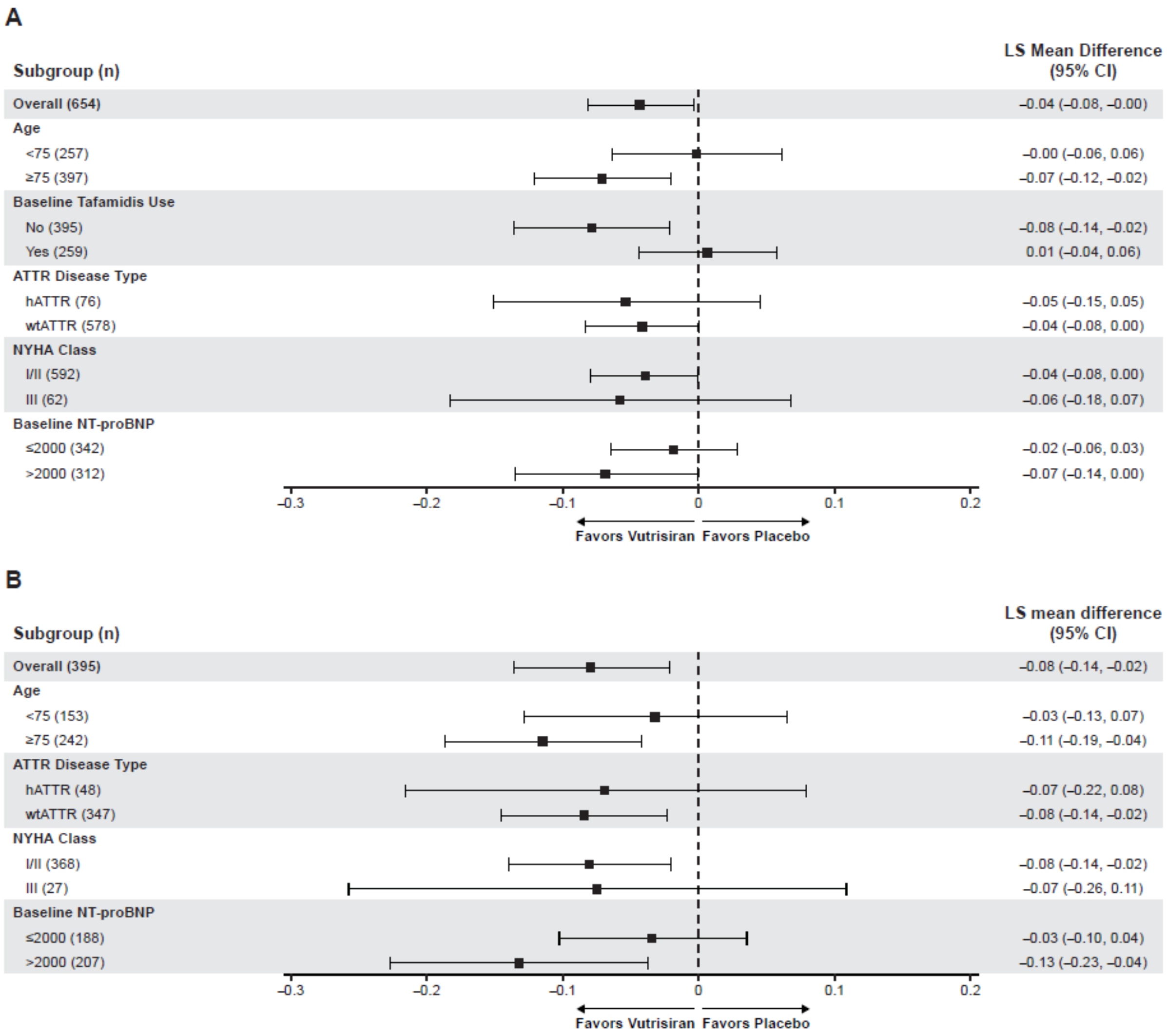

Supplementary Figure 2.

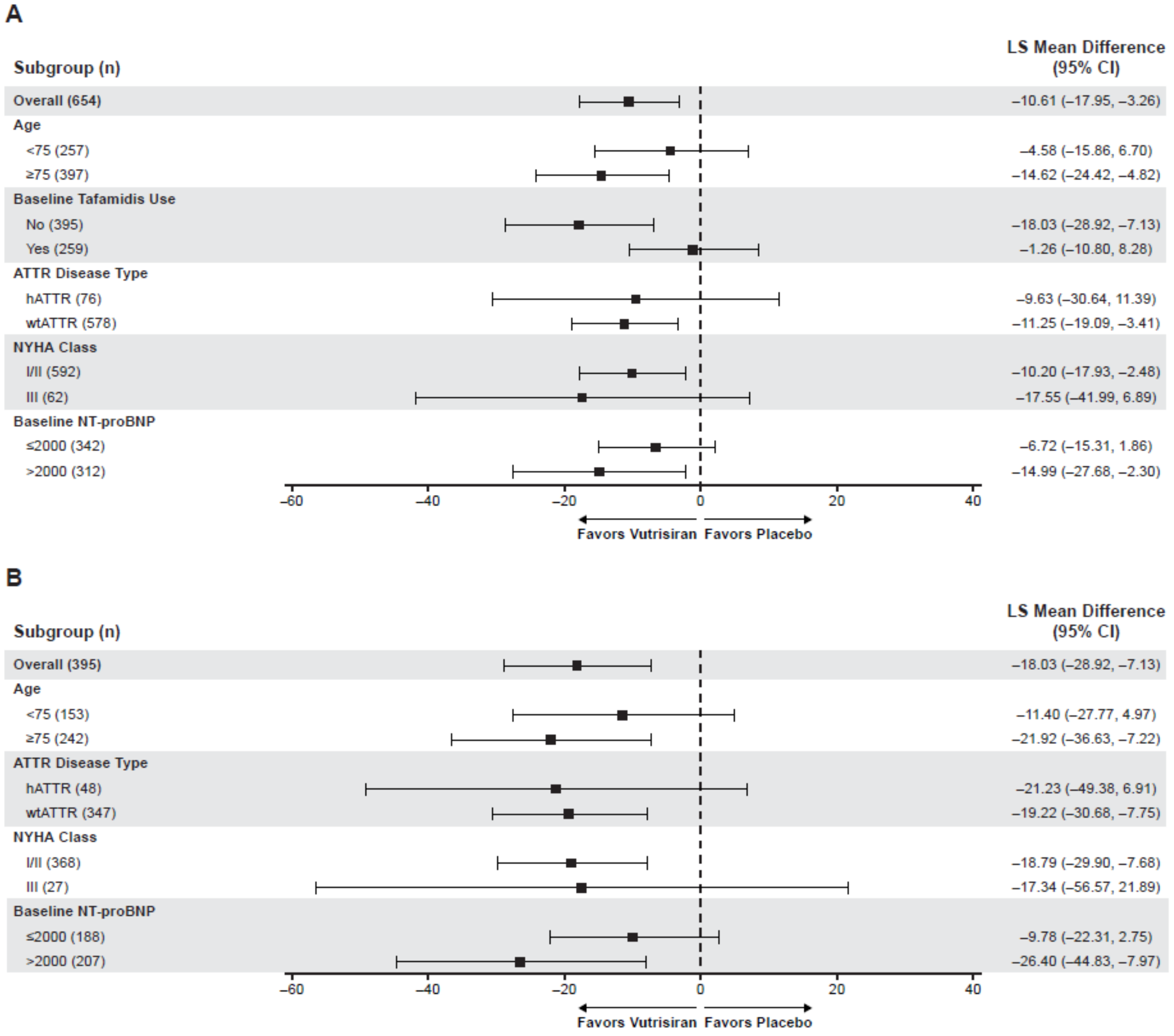

Supplementary Figure 3.

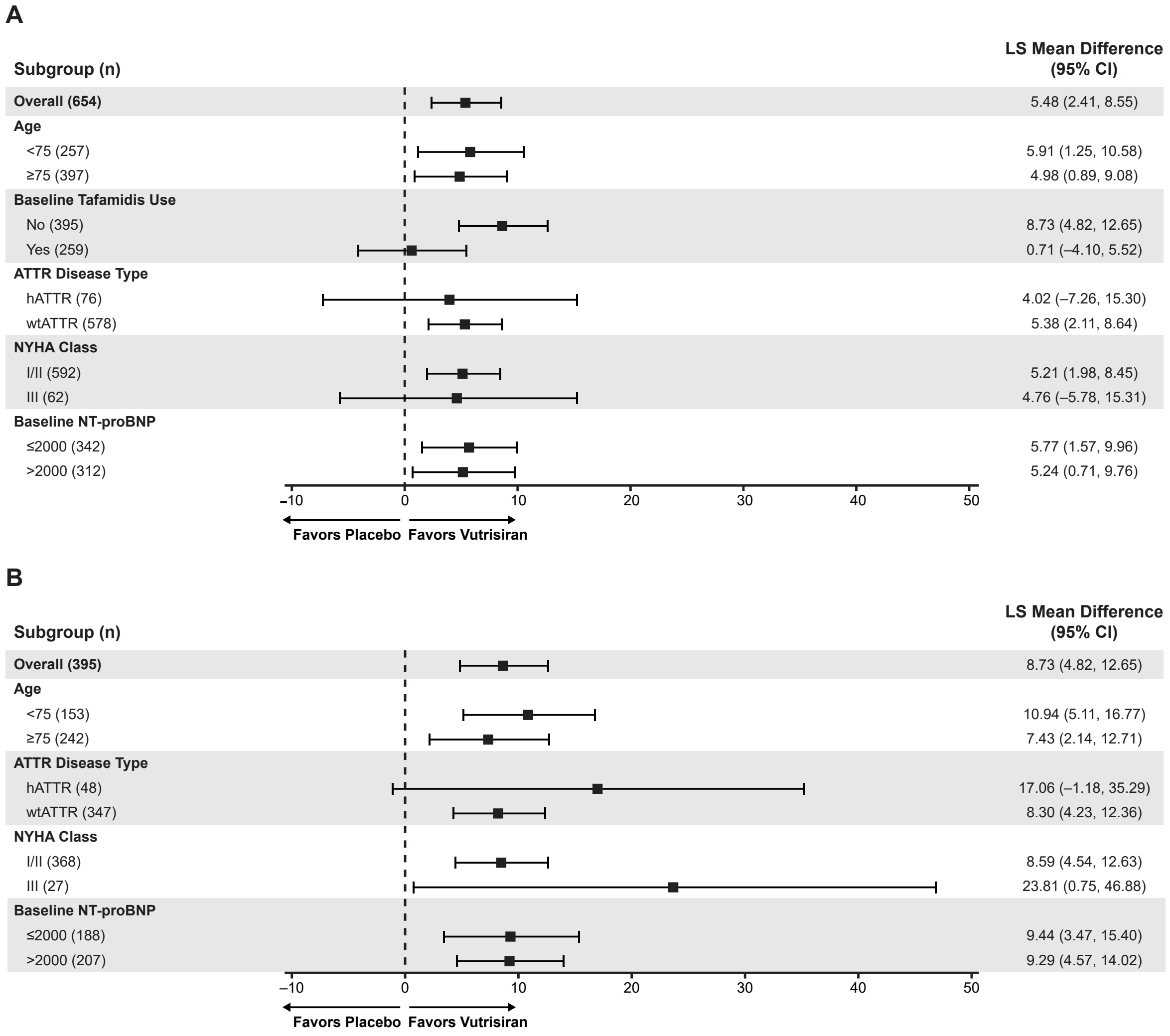

Supplementary Figure 4.

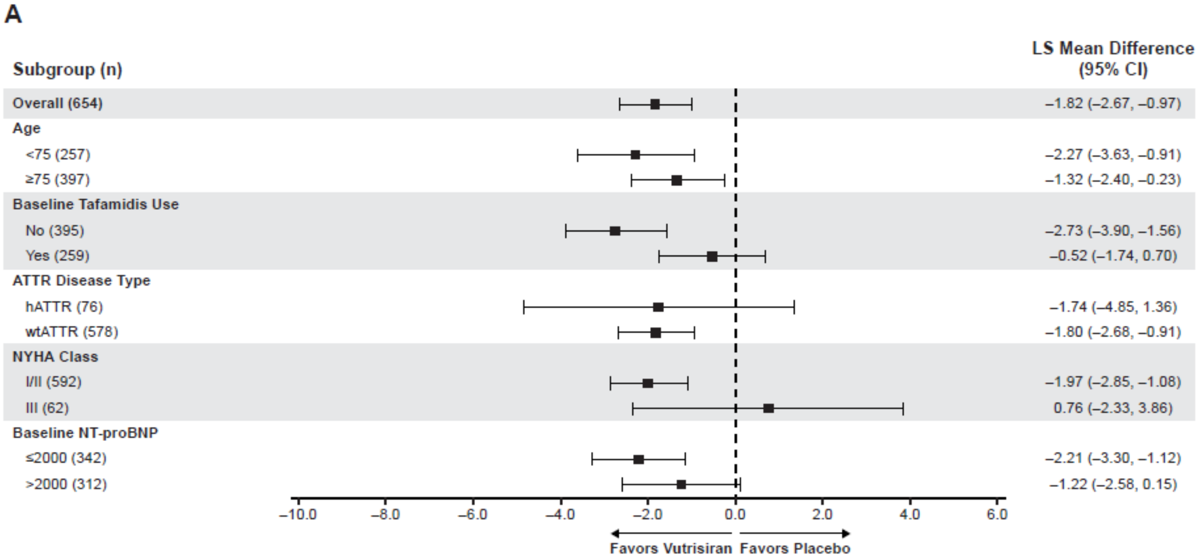

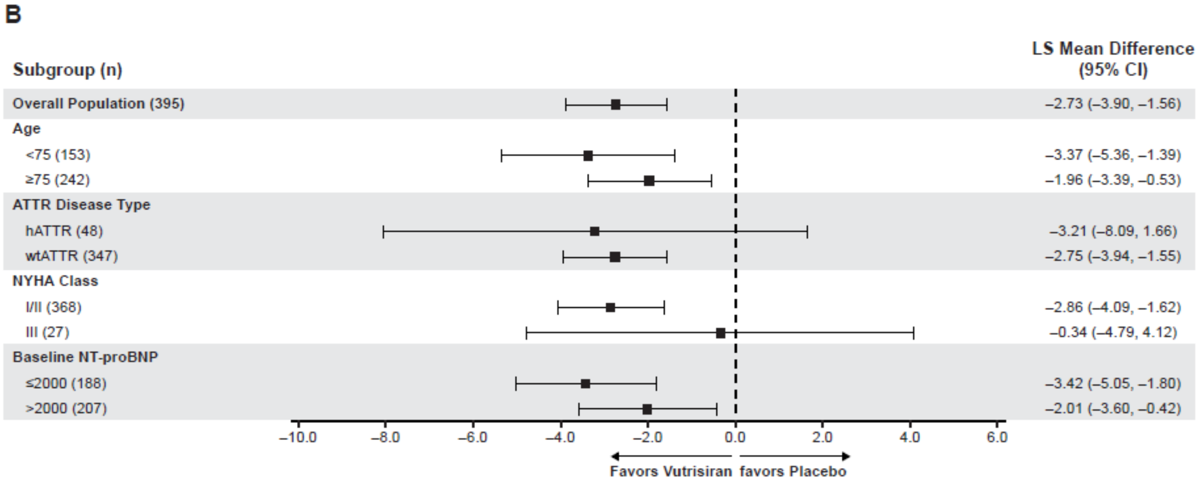

Supplementary Figure 5.

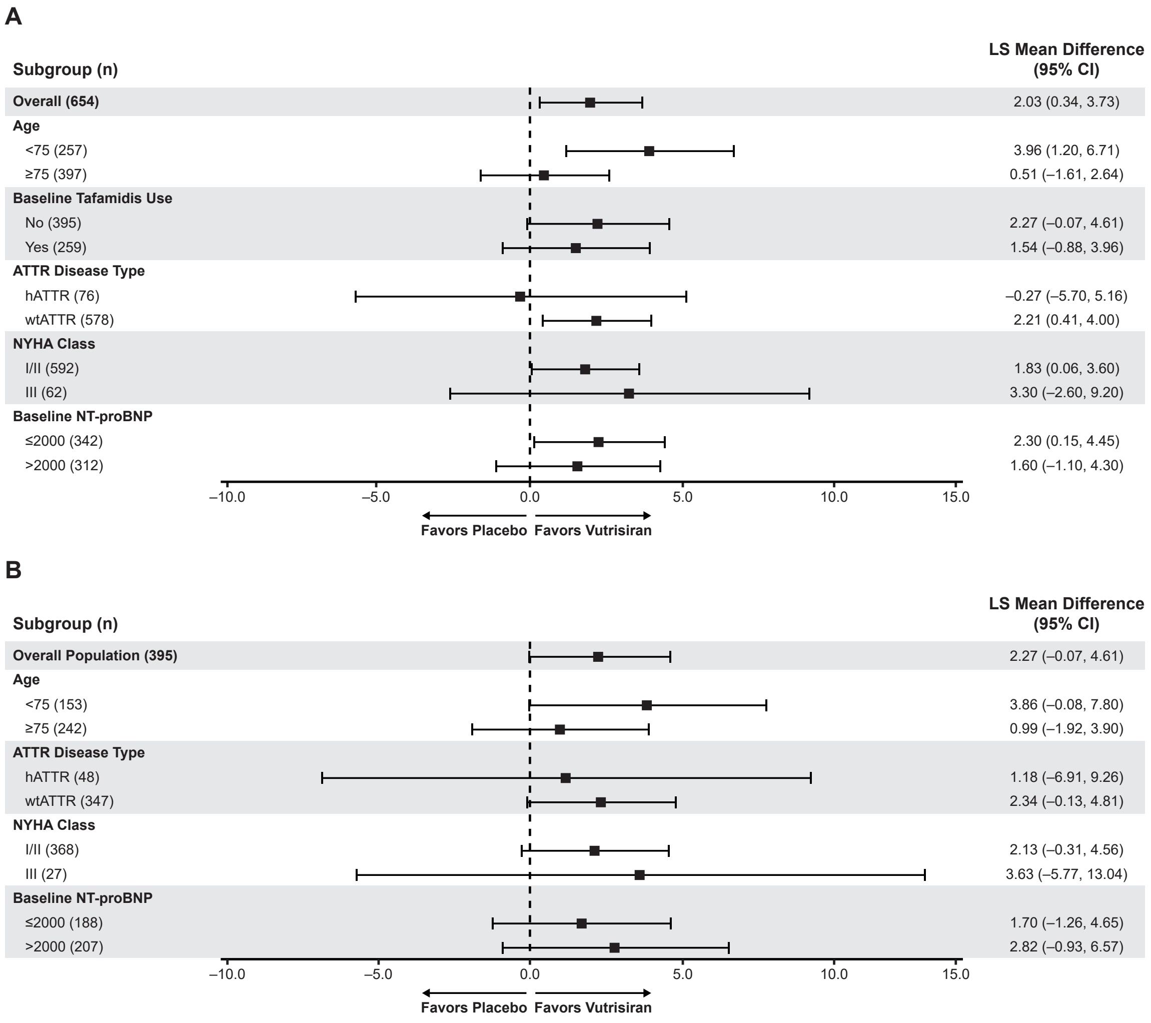

Supplementary Figure 6.

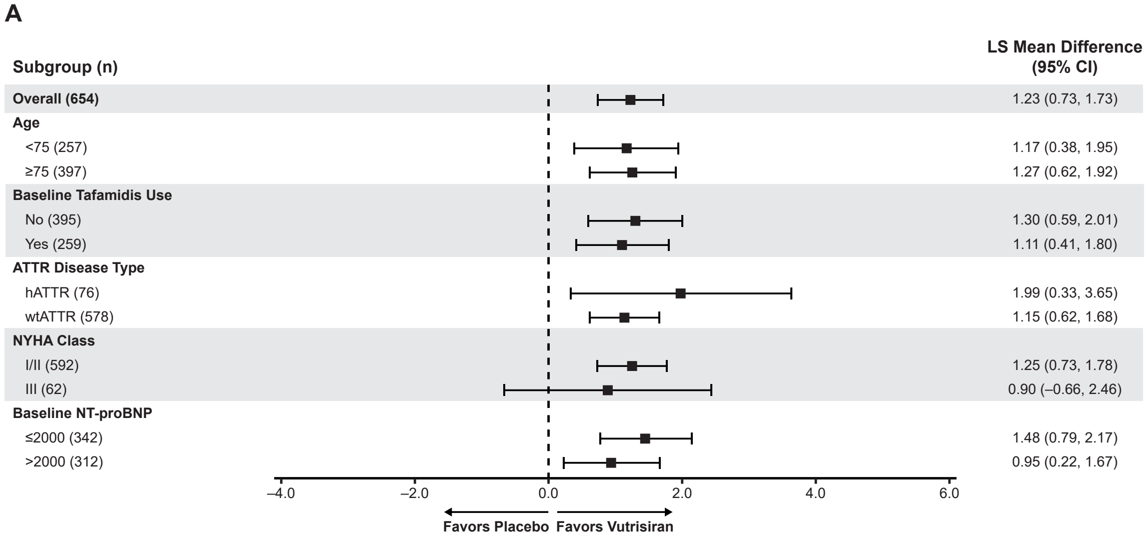

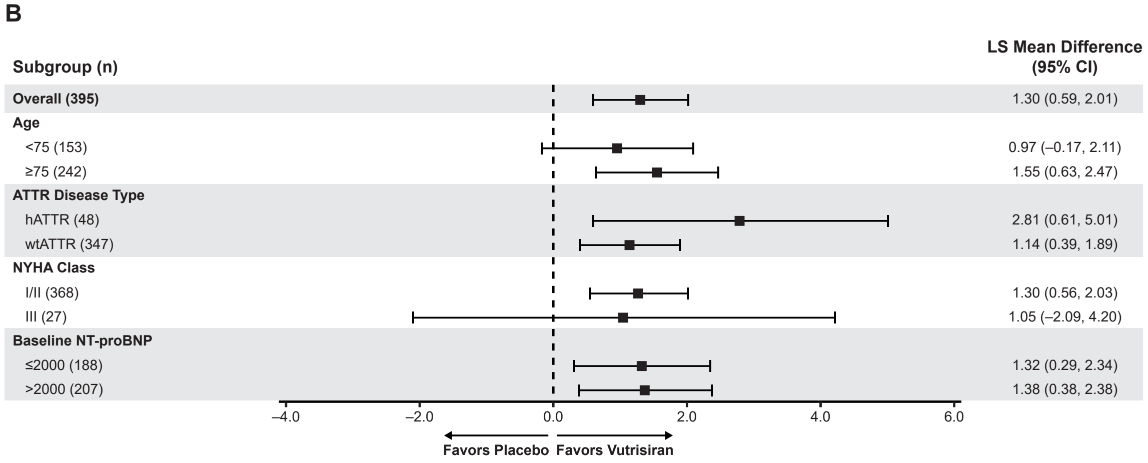

Supplementary Figure 7.

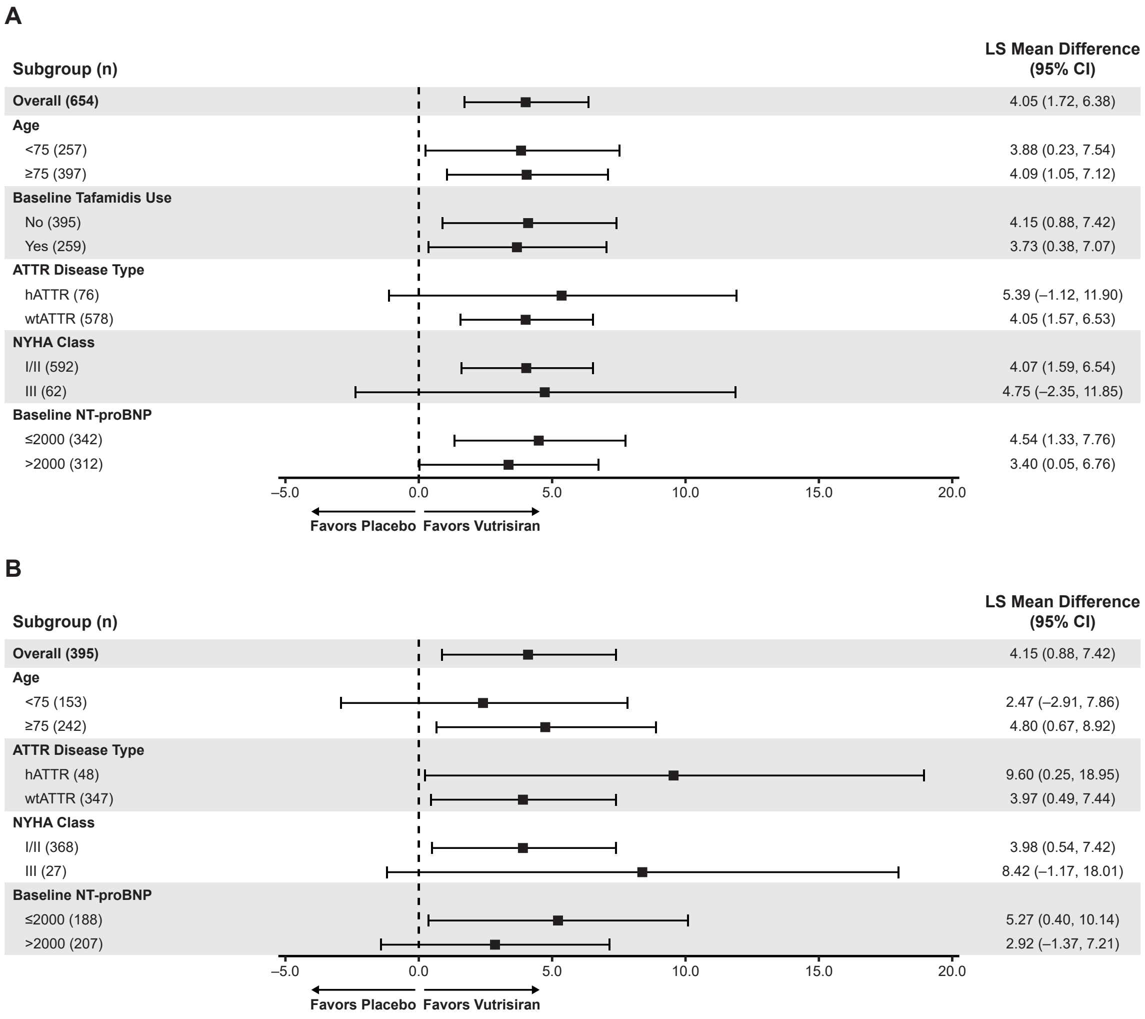

Supplementary Figure 8.

A

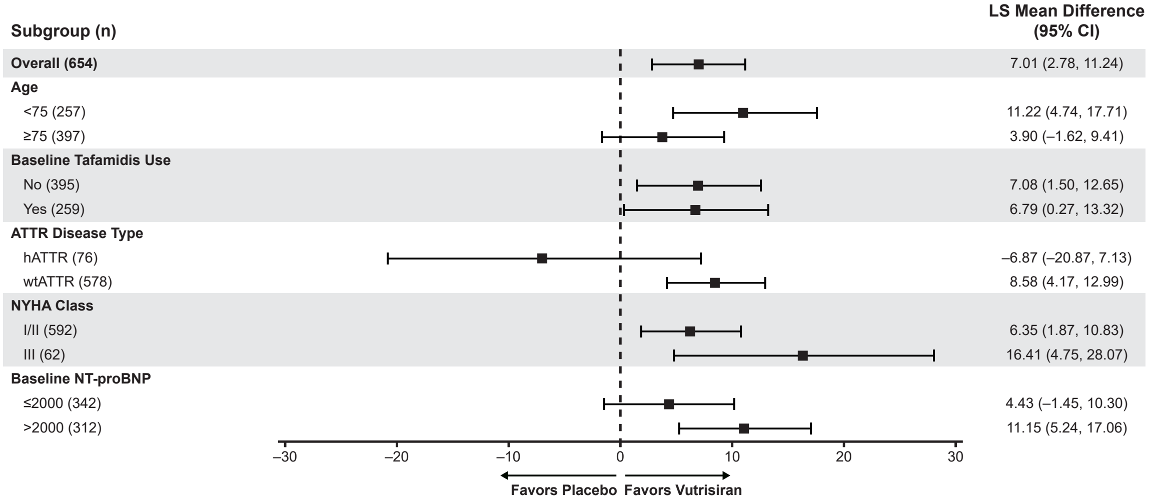

B

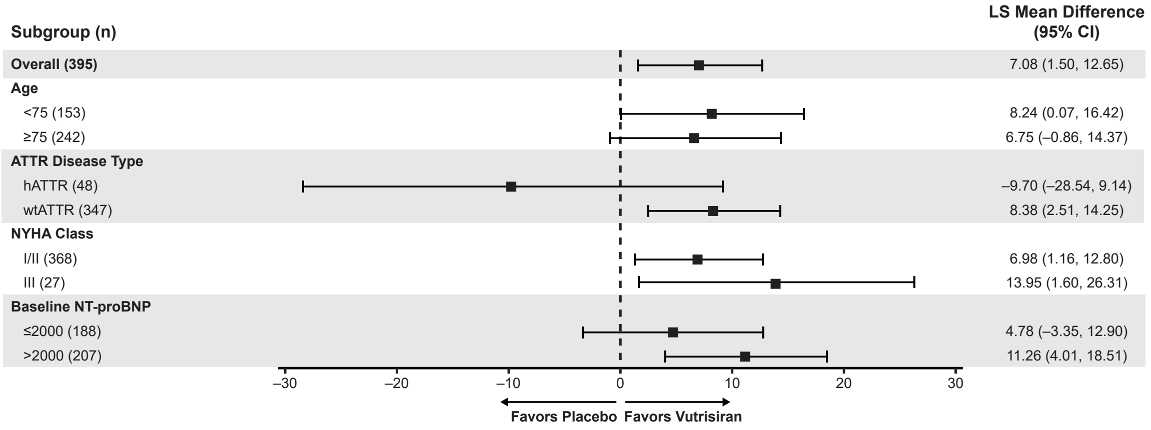

Supplementary Table

Supplementary Table 1. Missing Echocardiographic Variables at Baseline and Month 30.

	**Baseline**				**Month 30**			
	**Placebo**		**Vutrisiran**		**Placebo**		**Vutrisiran**	
	n	%	n	%	n	%	n	%
Number of patients who died before visit	0	0	0	0	61	18.6	51	15.6
Number of patients who discontinued before visit	0	0	0	0	21	6.4	12	3.7
Number of patients who are alive and in-study at visit	328	0	326	0	246	75	263	80.7
**Missing echocardiographic parameters**								
LV structure								
Mean LV wall thickness	4	1.2%	5	1.5%	107	32.6%	86	26.4%
LVEDD	8	2.4%	4	1.2%	114	34.8%	91	27.9%
LVEDV	8	2.4%	16	4.9%	107	32.6%	88	27.0%
LVESD	38	11.6%	40	12.3%	134	40.9%	110	33.7%
LVESV	10	3.0%	17	5.2%	113	34.5%	93	28.5%
LV mass index	10	3.0%	7	2.1%	117	35.7%	93	28.5%
LV systolic function								
LVEF	10	3.0%	17	5.2%	113	34.5%	93	28.5%
Absolute global longitudinal strain	0	0.0%	2	0.6%	101	30.8%	81	24.8%
Stroke volume	21	6.4%	17	5.2%	123	37.5%	97	29.8%
TDI lateral s'	12	3.7%	21	6.4%	113	34.5%	98	30.1%
TDI septal s'	7	2.1%	17	5.2%	113	34.5%	98	30.1%
LV diastolic function								
E/A ratio	157	47.9%	144	44.2%	222	67.7%	200	61.3%
E wave	7	2.1%	3	0.9%	112	34.1%	85	26.1%
A wave	156	47.6%	144	44.2%	220	67.1%	199	61.0%

Deceleration time	17	5.2%	10	3.1%	120	36.6%	95	29.1%
TDI lateral e'	12	3.7%	15	4.6%	110	33.5%	93	28.5%
TDI septal e'	8	2.4%	14	4.3%	114	34.8%	98	30.1%
Lateral E/e'	17	5.2%	17	5.2%	119	36.3%	97	29.8%
Septal E/e’	13	4.0%	16	4.9%	123	37.5%	101	31.0%
Average E/e’	20	6.1%	22	6.7%	125	38.1%	104	31.9%
Left atrial size and function								
LA diameter	13	4.0%	9	2.8%	121	36.9%	99	30.4%
LA volume index	3	0.9%	13	4.0%	104	31.7%	87	26.7%
TDI lateral a'	120	36.6%	131	40.2%	179	54.6%	166	50.9%
TDI septal a'	149	45.4%	160	49.1%	198	60.4%	190	58.3%
Right ventricle and pulmonary pressure								
RV free wall thickness	95	29.0%	102	31.3%	158	48.2%	155	47.5%
RV end-diastolic area	44	13.4%	52	16.0%	146	44.5%	133	40.8%
RV end-systolic area	46	14.0%	54	16.6%	155	47.3%	144	44.2%
Tricuspid annular systolic myocardial velocity (S')	10	3.0%	19	5.8%	123	37.5%	105	32.2%
TR velocity	56	17.1%	63	19.3%	145	44.2%	122	37.4%
Maximal IVC diameter	70	21.3%	76	23.3%	128	39.0%	115	35.3%

a’ – peak late diastolic mitral annular tissue velocity, A wave – peak late diastolic transmitral flow velocity, e’ – peak early diastolic mitral annular tissue velocity, E wave – peak early diastolic transmitral flow velocity, IVC – inferior vena cava, LA – left atrial, LV – left ventricular, LVEDD – left ventricular end-diastolic dimension, LVEDV – left ventricular end-diastolic volume, LVEF – left ventricular ejection fraction, LVESD – left ventricular end-systolic dimension, LVESV – left ventricular end-systolic volume, s’ – peak systolic mitral annular tissue velocity, TDI – tissue Doppler imaging, TR – tricuspid regurgitation.
