# Supplementary material for: Effects of vutrisiran on cardiac structure and function in patients with transthyretin amyloidosis with cardiomyopathy: secondary outcomes of the HELIOS-B trial
Source: Nat Med. 2025 Aug 6;31(10):3560–8. doi: 10.1038/s41591-025-03851-z (PMC12532587; doi:10.1038/s41591-025-03851-z)
Supplement: Supplementary file 2 — Reporting Summary [file 41591_2025_3851_MOESM2_ESM.pdf]

nature portfolio

Corresponding author(s): Dr Scott Solomon

Last updated by author(s): 05/16/2025

Reporting Summary

Nature Portfolio wishes to improve the reproducibility of the work that we publish. This form provides structure for consistency and transparency in reporting. For further information on Nature Portfolio policies, see our Editorial Policies and the Editorial Policy Checklist.

Statistics

For all statistical analyses, confirm that the following items are present in the figure legend, table legend, main text, or Methods section.

n/a	Confirmed	
☐	☒	The exact sample size (*n*) for each experimental group/condition, given as a discrete number and unit of measurement
☒	☐	A statement on whether measurements were taken from distinct samples or whether the same sample was measured repeatedly
☐	☒	The statistical test(s) used AND whether they are one- or two-sided<br>*Only common tests should be described solely by name; describe more complex techniques in the Methods section.*
☐	☒	A description of all covariates tested
☐	☒	A description of any assumptions or corrections, such as tests of normality and adjustment for multiple comparisons
☐	☒	A full description of the statistical parameters including central tendency (e.g. means) or other basic estimates (e.g. regression coefficient) AND variation (e.g. standard deviation) or associated estimates of uncertainty (e.g. confidence intervals)
☐	☒	For null hypothesis testing, the test statistic (e.g. *F*, *t*, *r*) with confidence intervals, effect sizes, degrees of freedom and *P* value noted<br>*Give P values as exact values whenever suitable.*
☒	☐	For Bayesian analysis, information on the choice of priors and Markov chain Monte Carlo settings
☒	☐	For hierarchical and complex designs, identification of the appropriate level for tests and full reporting of outcomes
☒	☐	Estimates of effect sizes (e.g. Cohen's *d*, Pearson's *r*), indicating how they were calculated

Our web collection on statistics for biologists contains articles on many of the points above.

Software and code

Policy information about availability of computer code

Data collection	Rave EDC version 2024.1.1. was used for case report form data collection. Echocardiographic measurements were performed using commercially available software (US2.ai versions 1.4 and 2.0).
Data analysis	Statistical analyses were performed using SAS version 9.4.

For manuscripts utilizing custom algorithms or software that are central to the research but not yet described in published literature, software must be made available to editors and reviewers. We strongly encourage code deposition in a community repository (e.g. GitHub). See the Nature Portfolio guidelines for submitting code & software for further information.

Data

Policy information about availability of data

All manuscripts must include a data availability statement. This statement should provide the following information, where applicable:

- Accession codes, unique identifiers, or web links for publicly available datasets
- A description of any restrictions on data availability
- For clinical datasets or third party data, please ensure that the statement adheres to our policy

Access to anonymized individual participant data that support these results is made available 12 months after study completion and not less than 12 months after the product and indication have been approved in the US and/or the EU. Access to data may be declined where there is likelihood a patient could be identified or other feasibility issue, where there is a potential conflict of interest, planned business activities or an actual or potential competitive risk. Data will be provided

contingent upon the approval of a research proposal and the execution of a data sharing agreement. Timeframes for data access may vary and can take up to 6 months or more. Requests for access to data can be submitted via the website www.vivli.org. Questions can also be directed to datasharing@alnylam.com.

Research involving human participants, their data, or biological material

Policy information about studies with human participants or human data. See also policy information about sex, gender (identity/presentation), and sexual orientation and race, ethnicity and racism.

Reporting on sex and gender	The n (%) of males/females was described overall and by randomized therapy. Sex was self-reported. Heterogeneity of treatment effect by sex was not evaluated due to the relatively low number of women enrolled in HELIOS-B, consistent with the reported demographic characteristics of patients with ATTR-CM. Given the male preponderance of patients with ATTR-CM, power to estimate treatment effects according to sex was limited.
Reporting on race, ethnicity, or other socially relevant groupings	The n (%) of each racial category (White, Asian, Black, Other) was described by randomized therapy. Race was self-reported.
Population characteristics	Patients 18–85 years of age with a diagnosis of either variant or wild-type ATTR-CM established on the basis of tissue biopsy or validated scintigraphy-based diagnostic criteria with evidence of cardiac involvement (interventricular septal wall thickness >12 mm on echocardiography) and a clinical history of symptomatic HF.<br>Median (interquartile range [IQR]) age was 77 years (45–85), 93% were male, 88% had wild-type ATTR-CM, 91% New York Heart Association functional class ≤ II and 67% National Amyloidosis Centre stage 1. Approximately 40% of patients were using tafamidis at baseline.
Recruitment	Participants were recruited from 87 sites across 26 countries. Participants were enrolled from academic/hospital-based or community health care facilities and per protocol could not have been hospitalized for cardiovascular reasons in the six weeks prior to randomization. All participants were required to meet specific inclusion and exclusion criteria, as specified in the study protocol, to be considered eligible for the trial and there was no self-selection. Effects of selection bias on the outcomes reported in this study were minimal given the randomized design.
Ethics oversight	The local ethics committee at each participating site approved the study protocol, and patients provided written informed consent in accordance with established guidelines. The complete list of committees that approved the HELIOS-B protocol is provided in the supplementary appendix.

Note that full information on the approval of the study protocol must also be provided in the manuscript.

Field-specific reporting

Please select the one below that is the best fit for your research. If you are not sure, read the appropriate sections before making your selection.

☒ Life sciences ☐ Behavioural & social sciences ☐ Ecological, evolutionary & environmental sciences

For a reference copy of the document with all sections, see nature.com/documents/nr-reporting-summary-flat.pdf

Life sciences study design

All studies must disclose on these points even when the disclosure is negative.

Sample size	Enrollment of 600 patients was planned and 654 patients were randomized, including 60% patients in the vutrisiran monotherapy group. Sample size was calculated based on the following assumptions such that the study would have approximately 80% power in both the overall population and the vutrisiran monotherapy group to detect a difference between treatment groups using a modified Andersen-Gill model with robust variance estimator, with a 2-sided alpha of 0.05:<br>- In the monotherapy subgroup, vutrisiran provides a 25% reduction in mortality rates and a 35% reduction in recurrent cardiovascular event rates compared with placebo over 30 months; 0.34 cardiovascular events per patient-year and 25% mortality rate at month 30 were assumed in the placebo group.<br>- In the background tafamidis subgroup, vutrisiran plus tafamidis provides a 10% reduction in mortality rates and a 15% reduction in recurrent cardiovascular event rates compared with placebo plus tafamidis over 30 months.<br>- A 9-month and 18-month delay to effect on cardiovascular events and death, respectively.<br>- In the vutrisiran monotherapy subgroup, approximately 20% of patients in both treatment arms add tafamidis anytime during the first 24 months on study.<br>- Cardiovascular events and mortality data will be collected up to 36 months and no more than 15% of patients in both treatment arms are lost to follow-up.
Data exclusions	Only patients with critical Good Clinical Practice violations or missing baseline echocardiograms were excluded.
Replication	Consistency in treatment effects for the primary and secondary endpoints was confirmed both in the overall study population and in the vutrisiran monotherapy group.<br>For this study, echocardiographic measurements were performed in triplicates whenever possible and were averaged. In patients with atrial fibrillation, measurements were repeated five times and were averaged. Lab-wide intra- and interobserver variability for key measures of cardiac structure and function have been previously reported; (1-3) amongst analysts involved in this study the coefficient of variation was ≤16% and intraclass correlation ≥0.72.

1. Shah AM, Shah SJ, Anand IS, et al. Cardiac structure and function in heart failure with preserved ejection fraction: baseline findings from the echocardiographic study of the Treatment of Preserved Cardiac Function Heart Failure with an Aldosterone Antagonist trial. Circulation: Heart Failure. 2014;7(1):104-115.
2. Shah AM, Cheng S, Skali H, et al. Rationale and Design of a Multicenter Echocardiographic Study to Assess the Relationship Between Cardiac Structure and Function and Heart Failure Risk in a Biracial Cohort of Community-Dwelling Elderly Persons. Circulation: Cardiovascular Imaging. 2014;7(1):173-181. doi:doi:10.1161/CIRCIMAGING.113.000736
3. Kraigher-Krainer E, Shah AM, Gupta DK, et al. Impaired systolic function by strain imaging in heart failure with preserved ejection fraction. J Am Coll Cardiol. Feb 11 2014;63(5):447-56. doi:10.1016/j.jacc.2013.09.052

Randomization

Patients were randomized in a 1:1 ratio to receive 25 mg of vutrisiran or placebo administered subcutaneously every 12 weeks. Randomization was stratified by: 1) baseline tafamidis use (yes versus no); 2) ATTR disease type (variant versus wild-type ATTR amyloidosis with cardiomyopathy); and 3) New York Heart Association Class I or II and age <75 years versus all other.

Blinding

HELIOS-B was a double-blind, placebo-controlled randomized clinical trial. Specifically, all investigators remained strictly blinded to treatment arm allocation during data collection and analysis. Furthermore, echocardiograms were reviewed by dedicated analysts blinded to clinical characteristics of study participants, treatment assignment, and temporal sequence.

Reporting for specific materials, systems and methods

We require information from authors about some types of materials, experimental systems and methods used in many studies. Here, indicate whether each material, system or method listed is relevant to your study. If you are not sure if a list item applies to your research, read the appropriate section before selecting a response.

Materials & experimental systems

n/a	Involved in the study	
☒	☐	Antibodies
☒	☐	Eukaryotic cell lines
☒	☐	Palaeontology and archaeology
☒	☐	Animals and other organisms
☐	☒	Clinical data
☒	☐	Dual use research of concern
☒	☐	Plants

Methods

n/a	Involved in the study	
☒	☐	ChIP-seq
☒	☐	Flow cytometry
☒	☐	MRI-based neuroimaging

Clinical data

Policy information about clinical studies
All manuscripts should comply with the ICMJE guidelines for publication of clinical research and a completed CONSORT checklist must be included with all submissions.

Clinical trial registration

NCT04153149

Study protocol

The study protocol and statistical analysis plan were published with the primary paper in NEJM (Fontana M, Berk JL, Gillmore JD, et al. Vutrisiran in Patients with Transthyretin Amyloidosis with Cardiomyopathy. N Engl J Med. Aug 30 2024;doi:10.1056/NEJMoa2409134).

Data collection

Participants in HELIOS-B were enrolled from December 2019 through August 2021 at 87 sites across 26 countries. Participants were enrolled from academic/hospital-based or community health care facilities and per protocol could not have been hospitalized for cardiovascular reasons in the preceding six weeks. A list of participating sites is included in the supplementary appendix.

Outcomes

The prespecified primary endpoint of HELIOS-B was a composite of all-cause death and recurrent cardiovascular events (defined as hospitalizations for cardiovascular causes or urgent visits for heart failure) during the double-blind period (up to 36 months). Clinical outcomes were adjudicated by an independent clinical events committee blinded to treatment assignment. Changes in cardiac structure and function (change from baseline in mean left ventricular wall thickness and global longitudinal strain) were prespecified as an outcome of interest in the study protocol and statistical analysis plan. Secondary outcomes including changes from baseline to month 30 in echocardiographic measures of cardiac structure and function (mean left ventricular wall thickness and global longitudinal strain) were pre-specified in the study protocol and statistical analysis plan.
Per protocol, certified sonographers at each site performed serial echocardiograms at baseline, month 12, 18, 24 and 30. Echocardiographic images were transferred to the Cardiovascular Imaging Core Laboratory (Brigham and Women's Hospital, Boston, MA, USA) and were evaluated by dedicated analysts blinded to clinical characteristics of study participants, randomized treatment assignment and temporal sequence. Echocardiographic measurements were performed in triplicate (five times for participants in atrial fibrillation) and were averaged. Measurements were performed using commercially available software (US2ai version 1.4 and 2.0, Singapore, Singapore) in accordance with American Society of Echocardiography guidelines.

Plants

Seed stocks

Report on the source of all seed stocks or other plant material used. If applicable, state the seed stock centre and catalogue number. If plant specimens were collected from the field, describe the collection location, date and sampling procedures.

Novel plant genotypes

Describe the methods by which all novel plant genotypes were produced. This includes those generated by transgenic approaches, gene editing, chemical/radiation-based mutagenesis and hybridization. For transgenic lines, describe the transformation method, the number of independent lines analyzed and the generation upon which experiments were performed. For gene-edited lines, describe the editor used, the endogenous sequence targeted for editing, the targeting guide RNA sequence (if applicable) and how the editor was applied.

Authentication

Describe any authentication procedures for each seed stock used or novel genotype generated. Describe any experiments used to assess the effect of a mutation and, where applicable, how potential secondary effects (e.g. second site T-DNA insertions, mosiacism, off-target gene editing) were examined.
